# Higher-order transient membrane protein structures

**DOI:** 10.1073/pnas.2421275121

**Published:** 2024-12-31

**Authors:** Yuxi Zhang, Hisham Mazal, Venkata Shiva Mandala, Gonzalo Pérez-Mitta, Vahid Sondoghdar, Christoph A. Haselwandter, Roderick MacKinnon

**Affiliations:** ^a^Laboratory of Molecular Neurobiology and Biophysics, The Rockefeller University, New York, NY 10065; ^b^HHMI, The Rockefeller University, New York, NY 10065; ^c^Max Planck Institute for the Science of Light, Erlangen 91058, Germany; ^d^Max-Planck-Zentrum für Physik und Medizin, Erlangen 91058, Germany; ^e^Department of Physics, Friedrich Alexander University Erlangen-Nürnberg, Erlangen 91058, Germany; ^f^Department of Physics and Astronomy, University of Southern California, Los Angeles, CA 90089; ^g^Department of Quantitative and Computational Biology, University of Southern California, Los Angeles, CA 90089

**Keywords:** self-assembly, higher-order transient structure, HOTS, membrane signaling, GPCR

## Abstract

This study shows that five membrane proteins—three GPCRs, an ion channel, and an enzyme—form self-clusters under natural expression levels in a cardiac-derived cell line. The cluster size distributions imply that these proteins self-oligomerize reversibly through weak interactions. When the concentration of the proteins is increased through heterologous expression, the cluster size distributions approach a critical distribution at which point a phase transition occurs, yielding larger bulk phase clusters. A thermodynamic model like that explaining micellization of amphiphiles and lipid membrane formation accounts for this behavior. We propose that many membrane proteins exist as oligomers that form through weak interactions, which we call higher-order transient structures (HOTS). The key characteristics of HOTS are transience, molecular specificity, and a monotonically decreasing size distribution that may become critical at high concentrations. Because molecular specificity invokes self-recognition through protein sequence and structure, we propose that HOTS are genetically encoded supramolecular units.

## Significance

Cells convey signals (information) across their membranes through the interactions of membrane proteins that communicate with each other, forming what are called pathways, akin to the components in an electronic circuit. But cell membranes are two-dimensional liquids in which diffusion dominates, raising the questions how do the components connect, and why do coexisting pathways not interfere with each other? This study shows that many membrane proteins self-assemble into higher-order transient structures (HOTS). Because HOTS are formed through specific protein interactions, they must be genetically encoded macromolecular units. In an accompanying paper, we show that HOTS connect a GPCR to an ion channel. HOTS can explain a dynamic connectivity of components in membrane signaling pathways.

Living cells sense their external environment through chemical processes that occur within and across the plasma membrane. For example, a G protein–coupled receptor in the plasma membrane binds to a molecule outside, causing a change in the receptor’s shape, converting it to a catalyst that splits G protein trimers inside (1, 2). The products of G protein trimer splitting, Gα and Gβγ, diffuse on the membrane surface where they bind to other membrane proteins—enzymes and ion channels—to regulate their function. The outcome of this sequence of chemical events is a change in the behavior of the cell. A given cell often will have multiple unique G protein pathways resident in its membrane, functioning simultaneously, and even sharing components, along with other signaling pathways that do not depend on G proteins (3, 4). The plasma membrane thus contains within it a network of signaling pathways that communicate information through chemical interactions to elicit unique cellular responses.

What determines connectivity of the components within a membrane signaling pathway, ensuring that it can function with fidelity and without interference from other pathways that coexist in the same membrane? In electronic devices, such connectivity is achieved through the fixed attachment of components that define a circuit. But in cells, the molecular components—the receptors, enzymes, ion channels, and G proteins—undergo constant thermal motion, diffusing rapidly within the two-dimensional liquid membrane. In this environment, where does the connectivity originate?

**Fig. 1.**
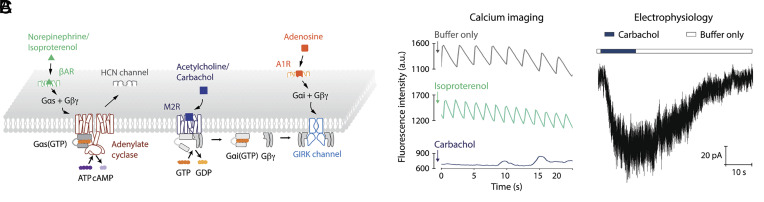
HL-1 cells contain β1AR and M2R signaling pathways. (*A*) Schematic of M2R, A1R, and β1AR signaling pathways in HL-1 cells. (*B*) Spontaneous calcium oscillations in confluent HL-1 cells are accelerated by isoproterenol and slowed by carbachol. Data are shown as the mean fluorescence over time of Fluo-8 AM loaded cells treated with buffer only, 10 μM isoproterenol or 10 μM carbachol. (*C*) HL-1 cells have the M2R-GIRK channel signaling pathway. Representative current trace measured in whole-cell mode showing the response of HL-1 cells to 10 μM carbachol. Voltage was held at –60 mV. Buffer conditions are described in *Materials and Methods*.

This question arose when we were faced with a puzzle concerning the activation of G protein–gated K^+^ (GIRK) channels by muscarinic type 2 (M2R) G protein–coupled receptors (GPCRs) in cardiac cells. Vagus nerve stimulation slows heart rate by causing M2R to split G protein trimers to generate Gβγ subunits, which bind to and open the GIRK channel (5–7) (Fig. 1*A*). Given the rate at which M2R is thought to generate Gβγ, and the low affinity of Gβγ for GIRK, it seemed that a single M2R could not generate enough Gβγ to activate GIRK even if the receptor and channel were bound together as a complex in the membrane (8, 9). Thus, we set out to describe the localization of M2R and GIRK channels on the plasma membrane of cardiac-derived HL-1 cells, in which the receptor and channel naturally reside and function (10, 11). We found that M2R exists in small clusters, and that quantitative features of the clusters implied an intriguingly simple principle for their formation. We extended our analysis to include five membrane proteins: three GPCRs, the GIRK channel, and the enzyme adenylate cyclase, all at natural levels of expression in HL-1 cells. Our findings are described in two papers.

This first paper examines the cluster size distributions of five membrane proteins in HL-1 cells, the behavior of these distributions upon overexpression in CHO cells, and, for M2R, some dynamical properties of these clusters. It has been well established that many membrane proteins form clusters (12). Several different mechanisms for cluster formation have been proposed, including assembly on protein scaffolds (13, 14), partitioning into regions of the membrane with favorable chemical and physical properties (15), and self-assembly of proteins (16–18). It is possible that all these mechanisms of cluster formation are important in biology. But a quantitative examination of clusters is needed with analysis in the context of theory. We present here evidence that five different membrane proteins in HL-1 cells self-oligomerize through weak yet specific interactions, resulting in the formation of higher-order transient structures (HOTS).

The second paper examines the potential biological role of HOTS in the M2R-GIRK signaling pathway (19). We define a property, dynamic connectivity, which we propose facilitates communication among components of a signaling pathway in an environment dominated by diffusion.

## Results

### Signaling in HL-1 Cells.

This study focuses on the mesoscale organization of membrane proteins that form pathways of signal communication in the plasma membrane. Except where specifically indicated, we present the density and distribution of proteins that are expressed endogenously, at physiological concentrations, in HL-1 cells (11). These cells, an immortalized line derived from the mouse, in culture behave like atrial cardiac cells, exhibiting rapid behavioral changes in response to neurotransmitters (Fig. 1), (7, 10, 20). Fig. 1*B* and movies show periodic oscillations of intracellular Ca^2+^ that change frequency in response to the β adrenergic receptor (β1AR) agonist isoproterenol, the M2R agonist carbachol, or the adenosine receptor (A1R) agonist adenosine (Fig. 1*B* and *SI Appendix*, Movies S1–S4). Three signaling pathways that regulate heart rate are outlined in Fig. 1*A*. In vivo, norepinephrine stimulates the β1AR to release Gαs, which activates adenylate cyclase (AC), increases cAMP levels, opens the HCN channel to depolarize the membrane potential, and ultimately speeds the heart rate (21, 22). Acetylcholine stimulates M2R to release Gβγ, which opens the GIRK channel to hyperpolarize the membrane potential and slow heart rate (2, 5, 8, 23–26). Adenosine, by acting on A1R, also slows heart rate by opening GIRK channels through the action of Gβγ (27, 28). Fig. 1*C* shows ion currents mediated by GIRK channels in an HL-1 cell when the M2R agonist carbachol is applied. This brief introduction to the signaling properties of HL-1 cells emphasizes that they are very much like natural cardiac cells, even contracting synchronously in a culture dish when grown to confluency.

### Five Membrane Proteins Form Clusters of Themselves.

**Fig. 2.**
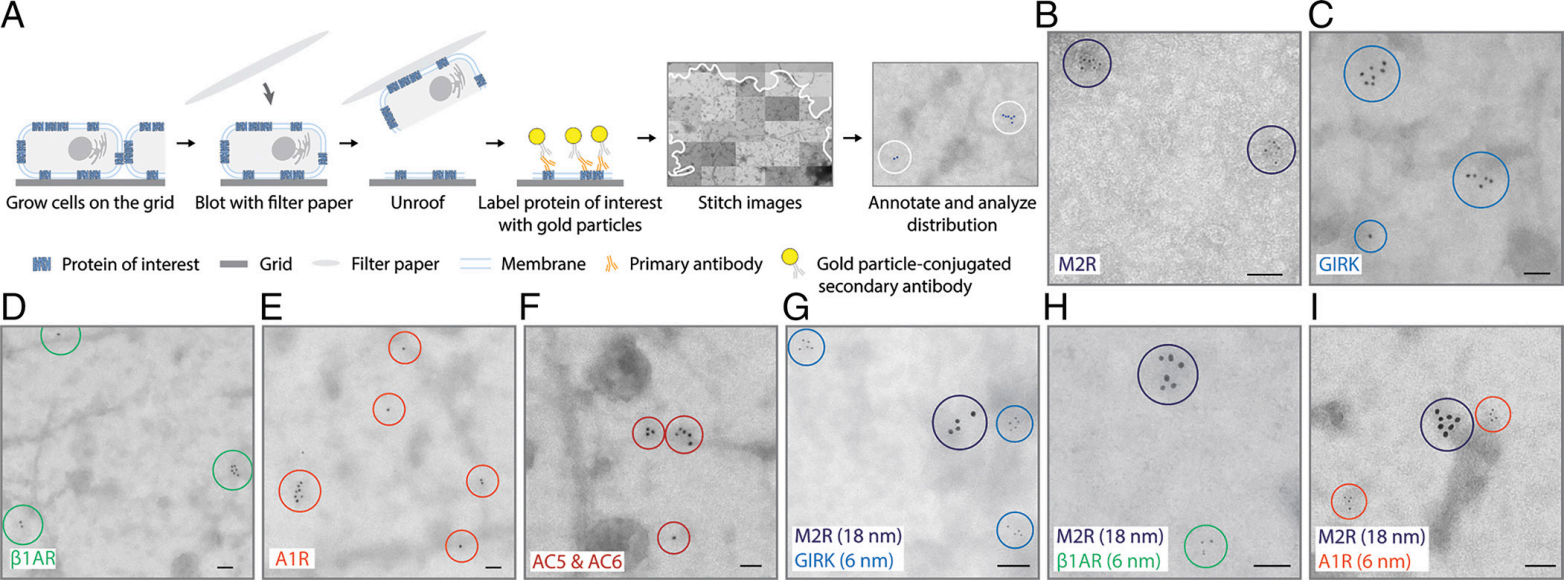
Electron microscope imaging of M2R, GIRK, β1AR, A1R, and AC distributions in HL-1 cells. (*A*) Schematic depicting the plasma membrane isolation (unroofing), gold particle labeling, and image analysis procedure. The unroofed membrane boundary is indicated by the solid curve. Circles indicate representative protein clusters. (*B*–*F*) M2R (*B*), GIRK (*C*), β1AR (*D*), A1R (*E*), and AC (*F*) all form clusters in HL-1 cells. Representative negative stain electron micrographs of M2R, GIRK, β1AR, A1R, and AC clusters are shown. M2R is labeled with 6 nm gold particles. GIRK, β1AR, A1R, and AC are labeled with 18 nm gold particles. Proteins are indicated by circles. (Scale bar, 100 nm.) (*G*–*I*) Protein clusters are self-specific. Representative negative stain electron micrographs with double-labeled M2R (18 nm) and GIRK (6 nm) (*G*), M2R (18 nm) and β1AR (6 nm) (*H*), and M2R (18 nm) and A1R (6 nm) (*I*) in HL-1 cells are shown. Circles with different colors indicate different proteins. (Scale bar, 100 nm.)

While the M2R-GIRK pathway and especially M2R is at the center of this study, we examined the membrane disposition of five proteins depicted in Fig. 1*A*: M2R, β1AR, A1R, GIRK, and AC. Fig. 2*A* outlines the procedure developed to isolate sheets of HL-1 plasma membranes by unroofing cells on electron microscope (EM) grids (29). After washing, membranes were labeled with a specific primary antibody and a gold particle-conjugated secondary antibody. Many primary antibodies were purchased and screened to identify ones that bound specifically and with low background binding, as described in *Materials and Methods*. The five proteins were labeled separately, and M2R-GIRK, M2R-β1AR, and M2R-A1R in pairs with distinguishable gold particles. Images were taken using an EM and stitched together to form a montage consisting of a membrane sheet from one or more cells with labels for analysis.

All five proteins occurred in small clusters of two or more, as defined in *SI Appendix*, Fig. S1, in addition to monomers (Fig. 2 *B*–*F*). The paired label experiments show that M2R, βA1R, A1R, and GIRK mostly form clusters only of themselves (Fig. 2 *G*–*I*). Because the primary antibody against M2R was derived from the rat and the primary antibody against the remaining proteins from the rabbit, all possible pairs could not be tested. However, the results suggest that proteins are segregated into protein type-specific clusters. We return to this specificity after considering other properties of these small clusters. With M2R, we analyzed clusters using negative stain EM and cryogenic EM (cryo-EM). Because the results were similar and negative stain EM is faster owing to microscope availability, we used the negative stain method except where indicated (*SI Appendix*, Fig. S2). To exclude the possibility that the clusters represent vesicles containing labeled proteins attached to the cytoplasmic surface, with M2R we made cryo-EM tomographic measurements and found that clusters are approximately planar, as expected if the proteins are embedded in the plasma membrane (*SI Appendix*, Fig. S3).

We note that clusters defined by gold particles tend to occur within larger, dark background patches on negative stain micrographs (Fig. 2). These patches are “protein-rich islands” that are crowded with many kinds of proteins (30, 31). Later in this paper, we explain why we think the protein type-specific clusters, the subject of this study, occur within the protein-rich islands.

### The Monotonically Decreasing Cluster Size Distribution.

**Fig. 3.**
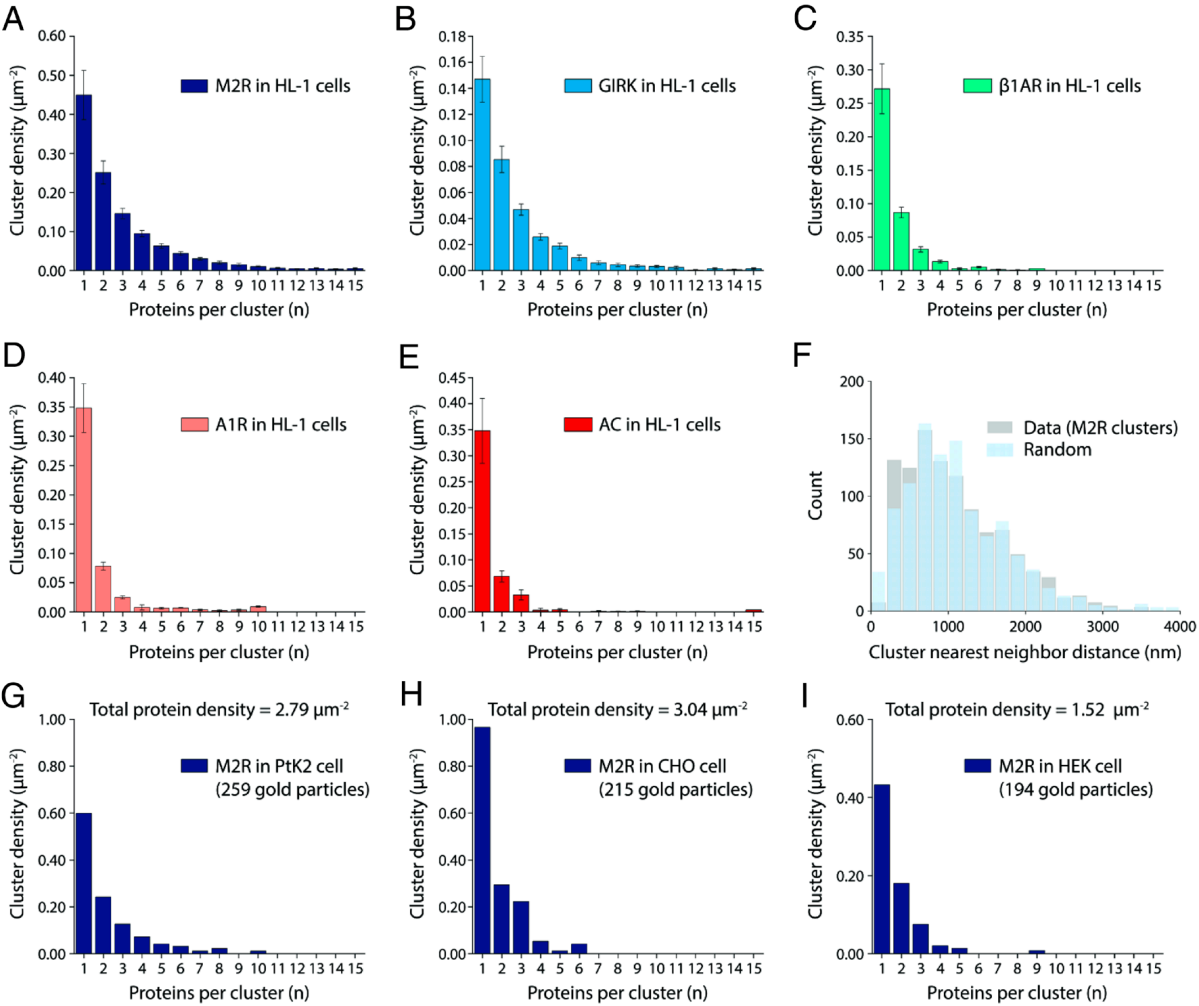
Analysis of M2R, GIRK, β1AR, A1R, and AC distributions in cell membranes. (*A*–*E*) The size distributions of M2R (*A*), GIRK (*B*), β1AR (*C*), A1R (*D*), and AC (*E*) clusters in HL-1 cells decrease monotonically with increasing cluster size n. M2R is labeled with 6 nm gold particles. GIRK, β1AR, A1R, and AC are labeled with 18 nm gold particles. The total particle densities are 4.3 µm^−2^ for M2R, 1.3 µm^−2^ for GIRK, 1.2 µm^−2^ for β1AR, 1.1 µm^−2^ for A1R, and 0.9 µm^−2^ for AC. For each protein, nonspecific gold particle labeling was estimated from CHO cells without heterologous expression and subtracted using the same reagents. Data represent means and SE from n EM montages: n = 13 for M2R (12,773 gold particles analyzed), n = 15 for GIRK (4,149 gold particles analyzed), n = 11 for β1AR (3,491 gold particles analyzed), n = 12 for A1R (2,636 gold particles analyzed), and n = 5 for AC (1,312 gold particles analyzed). (*F*) M2R clusters are randomly distributed on the surface of HL-1 cells. Nearest neighbor distance histograms of M2R cluster centroids (gray) and randomly distributed M2R clusters (blue) are shown. Clusters are defined so as to comprise at least three gold particles. Cluster centroids are calculated by averaging the x and y coordinates of all gold particles in each cluster. In the randomized case, the number of generated coordinates matches the number of cluster centroids, and the generated coordinates are randomly distributed over the regions of unroofed cells. (*G*–*I*) Cluster size distributions of M2R heterologously expressed in PtK2 (*G*), CHO (*H*), and HEK (*I*) cells decrease monotonically. Proteins are labeled with 18 nm gold particles. Nonspecific gold particle labeling was estimated from CHO cells without heterologous expression and subtracted. The total protein densities of M2R in the cell and the numbers of gold particles being analyzed are indicated. Each panel represents the result of one unroofed membrane.

Gold labels were identified in montages using the machine learning-based Dragonfly software package after training with a smaller dataset consisting of user selected gold particles (32). The graphs in Fig. 3 *A*–*E* show for each of the five proteins the density (μm^−2^) of monomers, dimers, trimers, etc., defined as in *SI Appendix*, Fig. S1. These are the cluster size distributions for each protein studied in HL-1 cells; size refers to the number *n* of proteins in a cluster. Cluster size distributions are monotonically decreasing for all five proteins, meaning monomers are most prevalent, next dimers, then trimers, etc., in decreasing prevalence. The M2R cluster size distribution in Fig. 3*A* is derived from 13 montages and more than 12,000 proteins identified using a 6 nm gold-conjugated secondary antibody and negative stain imaging. In *SI Appendix*, Fig. S2 we show M2R cluster size distributions using an 18 nm gold-conjugated secondary antibody with either negative stain imaging (from 5 montages) or cryo-EM imaging (from 6 montages). The M2R cluster size distributions from HL-1 cells measured with different-sized gold particles and by negative stain and cryo-EM are qualitatively similar. For quantitative analysis of the size distribution, we use the largest dataset, that with 6 nm gold labels, and negative stain imaging.

Fig. 3*F* graphs the distribution of nearest neighbor distances separating the centers of clusters of M2R. This distribution shows that M2R clusters are randomly distributed on the cell surface. A similar trend is observed for the other four proteins studied (*SI Appendix*, Fig. S4).

To examine whether clusters with monotonically decreasing size distributions are unique to native expression in HL-1 cells, M2R was expressed in three different cell lines, PtK2, CHO, and HEK, to mean densities close to that in HL-1 cells. Clusters with monotonically decreasing size distributions were observed in all cells (Fig. 3 *G*–*I*). Thus, the ability of M2R to form clusters is independent of cell type.

We measured M2R in HL-1 cells by a separate method that did not depend on the use of antibodies or gold labels. Specifically, we employed cryogenic optical localization in 3D (COLD) which can reach Angstrom resolution (33, 34). Here, HL-1 cells were grown on an electron microscope grid (*SI Appendix*, Fig. S5A) and were subsequently labeled with the fluorescent M2R antagonist ATTO655-telenzepine. The small size of this label permits an estimate of the spacing between individual proteins inside a cluster, which the antibody labeling method does not.

**Fig. 4.**
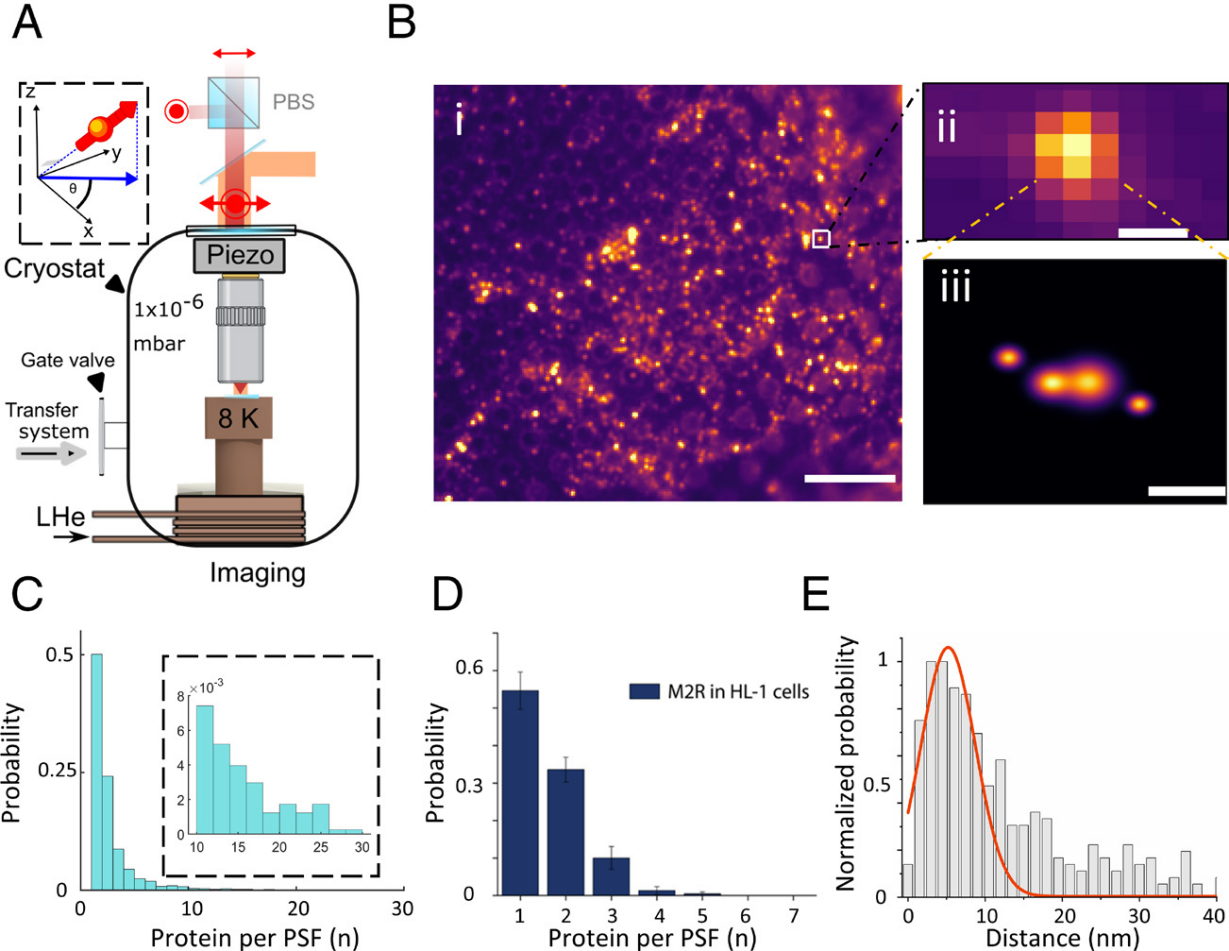
Cryogenic optical microscope imaging and analysis of the M2R distribution in HL-1 cells. (*A*) Schematic of the cryogenic optical microscope operating at 8 K. The setup was built to allow the transfer of vitrified biological samples under high vacuum and cryogenic temperature conditions. The microscope operates in polarization detection mode which allows us to resolve the random but fixed polarization states of each individual fluorophore at cryogenic temperature (inset). The number of polarizations per PSF indicates the number of molecules within the diffraction-limited spot. (*B*, *i*) Fluorescence image of vitrified, unroofed HL1 cells recorded with the cryo-microscope described in (*A*). (Scale bar, 10 μm.) (*B*, *ii*) Exemplary fluorescence signal within one PSF. The image in B(*i*–*ii*) is a sum of 5,000 frames. (Scale bar, 500 nm.) (*B*, *iii*) Having identified the polarization state over time (*SI Appendix*, Fig. S5D), we generate superresolved images by identifying the coordinates of each polarization state corresponding to a fluorophore. The extent of the spot in each localization represents the localization precision. (Scale bar, 10 nm.) (*C*) Distribution of the fluorescence intensities within a diffraction-limited PSF. The inset shows a close-up of the histogram tail (N = 4043). (*D*) Size distribution of the number of molecules per PSF based on the polarization time trace analysis, (N = 3620). (*E*) Distance histogram showing the distribution of distances between molecules within the diffraction-limited spot, obtained from the two-dimensional superresolved data with localization precision below 1 nm. The main peak of the distribution was fitted with a single Gaussian yielding a mean distance of 5.2 nm as an estimate for the most probable nearest neighbor distance.

The cells were unroofed, shock-frozen in liquid ethane, and imaged at 8 K in a home-built optical microscope housed in a liquid helium flow cryostat (Fig. 4*A*). Fig. 4 *B, i* displays an exemplary fluorescence image over a field of view of 51 × 45 μm^2^, consisting of isolated bright spots with some spatial distribution and a fairly large variation in intensity, down to the fluorescence of single fluorophores. The nearest neighbor distance between the PSFs, shown in *SI Appendix*, Fig. S5B, matches the one obtained from EM measurements (Fig. 3*F*). Most spots in the field correspond to diffraction-limited point-spread functions (PSFs), as shown in Fig. 4 *B, ii*. The histogram in Fig. 4*C* plots the distribution of the fluorescence signal recorded from such PSFs normalized to the average fluorescence of a single fluorophore (compare the signal from a large cluster versus a single molecule in *SI Appendix*, Fig. S5C). The data display a monotonic decrease in the number of proteins per PSF, like that measured with gold-labeled antibodies.

COLD takes advantage of the quantized fluorescence intensity or polarization that results from photoblinking of several fluorophores within a PSF to localize and superresolve them beyond the diffraction limit. By monitoring the polarization time trace, we analyzed and decomposed the recorded in-plane dipole orientation into *n* polarization states (Fig. 4*A*, *Inset* and *SI Appendix*, Fig. S5D), (34). The x–y position of the fluorophore, corresponding to each identified polarization state, was then determined, and a two-dimensional resolved image was reconstructed (Fig. 4 *B, iii*). Fig. 4*D* again confirms that the distribution of the number of the polarization states per PSF yields a monotonically decreasing pattern. More importantly, the method provides sufficiently high resolution to assess the distribution of the distances between individual M2R proteins within a small cluster, a quantity that is not accessible to the immunolabel method. To do this, we selected particles for which localization precision was better than 1 nm (*SI Appendix*, Fig. S5E) and calculated pairwise distance histograms. We find a main peak centered at 5.2 nm, close to the diameter of M2R, indicating that proteins are closely packed inside a cluster (Fig. 4*E*). The distribution in Fig. 4*E* also points to larger interprotein distances, albeit with substantially reduced probability beyond about 20 nm. We verified that relaxing the selection criterion from a localization precision of 1 nm to 3 nm does not change the outcome significantly (*SI Appendix*, Fig. S5F).

We note that the total density of M2R in the membrane cannot be accurately determined by COLD studies because the border of the cell membrane sheet is not as easy to identify in fluorescence microscopy as it is in negative-stain EM. However, COLD provides our best data on the relative locations of M2R within a cluster. The gold label method provides our most quantitative data on the size distribution of clusters. We next consider the cluster size distribution in greater detail and provide a potential origin for its monotonically decreasing form.

### Possible Mechanisms of Cluster Formation.

#### Attractor mechanisms.

**Fig. 5.**
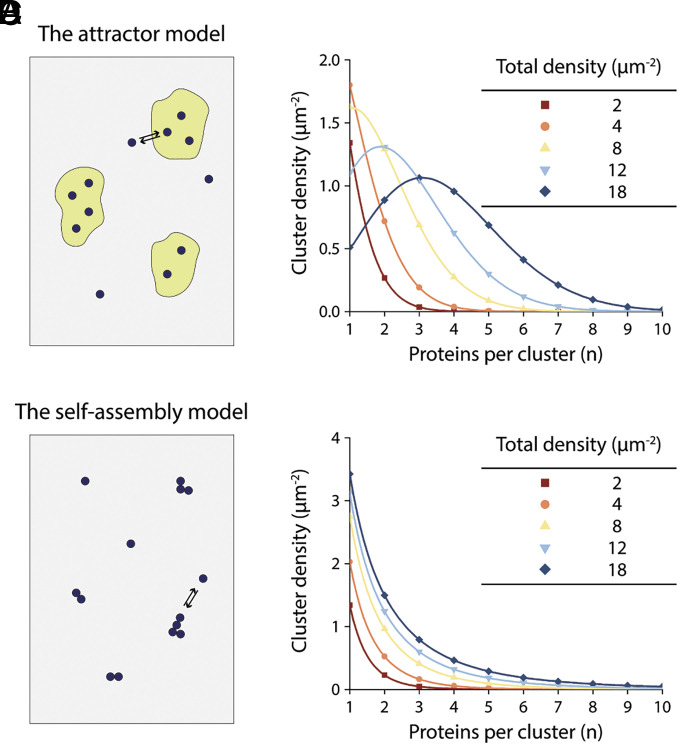
Predicted cluster size distributions of an attractor model and a self-assembly model. (*A*) Cartoon depicting an attractor model. The membrane is indicated by the gray area. Yellow patches are regions that attract a specific membrane protein. (*B*) Cluster size distributions for proteins at the indicated total densities predicted by the attractor model. The curves correspond to Eq. **9** in *SI Appendix*, Appendix 1 with $\begin{array}{c} K_{a}=200\;\mu m^{2},\;m_{\mathrm{tot}}=5.0\;\mu m^{-2},\;\;\text{and}\;\text{the}\;c_{\mathrm{tot}}\;(\mu m^{-2}) \end{array}$ indicated in the legend. (*C*) Cartoon depicting the self-assembly model. The membrane is indicated by the gray area. Specific proteins represented as black circles oligomerize reversibly. (*D*) Cluster size distributions for proteins at the indicated total densities predicted by the self-assembly model described in the main text. Curves correspond to Eq. **5** with $\Delta G_{\begin{array}{c} mon\;to\\ bulk \end{array}}^{0}=-2.0\;RT$, $A_{0}=0.04\;\mu m^{2}$, and the $c_{\mathrm{tot}}\;(\mu m^{-2})$ in the legend.

Scaffold proteins at the membrane surface are thought to bind specifically to certain membrane proteins (14). If scaffolds are localized at discrete sites on the cell membrane, for example at postsynaptic patches, then membrane proteins that bind favorably to them will become concentrated, i.e., clustered, at these sites. Another idea is that localized regions of a cell membrane, sometimes called rafts, might contain unique lipid compositions that are attractive to certain membrane proteins (35, 36). And yet another possibility is that localized variations in membrane thickness or curvature could serve to cluster membrane proteins (15). It seems possible that all these mechanisms are important in cell membranes to organize proteins in various circumstances. These different mechanisms operate on a common principle; they all attract proteins to particular membrane regions, resulting in preferred locations and sizes of protein clusters set by some external mechanism (Fig. 5*A*). Scaffolds have a finite number of sites and are therefore saturable, whereas the other mechanisms may be less so, but still, these are all attractors. Attractors predict cluster size distributions that are peaked (Fig. 5*B*). We provide a derivation of this result in *SI Appendix*, Appendix 1 for a particular model of a nonsaturable attractor, but the conclusion is easily generalized and is intuitive to understand. Attractors essentially partition “attracted” proteins into a region, giving rise to a Poisson-like size distribution (37). In the low concentration (of attracted protein) regime, where many attractors are empty (and those with proteins contain very few copies), the distribution can be monotonically decreasing, however, in a way that is quantitatively inconsistent with the distributions observed in this study. Moreover, we show later that the cluster size distributions of all five proteins remain monotonically decreasing as the concentration of protein is increased. The important point is, attractor mechanisms in general predict peaked distributions if the concentration of attracted proteins is sufficiently high. While some membrane proteins may cluster with peaked distributions, those in this study under the conditions we observe them do not.

#### Reversible oligomerization of weakly interacting proteins: HOTS.

Imagine if instead of being attracted to a particular place on the membrane, a protein recognizes and binds to itself through weak interactions, and thus forms transient oligomers (Fig. 5*C*). Importantly, the resulting size distribution of protein clusters is then an emergent property of the proteins forming the clusters, and not driven by some external attractor. The cluster size distribution for this scenario is derivable through reversible aggregation theory, which was developed long ago to explain certain self-assembly phenomena such as micellization of surfactants (38–43). When it was developed, reversible aggregation theory mostly focused on the occurrence of a phase transition, where “monomers” give rise to larger, more complex structures (micelles), because that is what could be measured. In our experiments, the focus is on the “monomers,” which are not just monomers, but a distribution of nmers that we observe directly. As far as we know, reversible aggregation theory has, until now, not been used to understand and predict quantitatively measured *nmer* distributions of proteins or any other molecules. We therefore give key steps of the theory here, and a derivation in *SI Appendix*, Appendix 2. The model is idealized; its purpose is to capture the general properties of the nmer (cluster) size distribution, to examine whether the measured membrane proteins behave according to this simple mechanism of cluster formation.

The idealized system is a planar surface represented, for illustration, as a two-dimensional grid with $N_{\mathrm{grid}}$ positions on which $N_{x}$ proteins of species x undergo a random walk. The grid has a constant area equal to $A_{0}N_{\mathrm{grid}}$, where $A_{0}$ is the unit grid area. For the monomer to nmer oligomerization reaction, n mon⇌1 nmer, at equilibrium $nG_{\mathrm{mon}}=G_{\mathrm{nmer}}$, where $G_{x}$ is the Gibbs free energy of x. When $N_{\mathrm{grid}}\gg N_{x}$, $G_{x}=G_{x}^{0}+RT\;\text{log}\left(\frac{N_{x}}{N_{grid}}\right)$, where $G_{x}^{0}$ is the standard Gibbs free energy of x. Therefore,

[1] $$\begin{array}{c} \Delta G_{\begin{array}{c} mon\;to\\ nmer \end{array}}^{0}\;\equiv G_{\mathrm{nmer}}^{0}-n\;{\text{G}}_{\mathrm{mon}}^{0}\\ \;=RT\;\text{log}\;\left[\frac{{\left(\frac{N_{mon}}{N_{grid}}\right)}^{n}}{\frac{N_{nmer}}{N_{grid}}}\right]\to RT\;\text{log}\left[\frac{{\left(A_{0}c_{\mathrm{mon}}\right)}^{n}}{A_{0}c_{nmer}}\right], \end{array}$$

where for the last identity we have noted that the monomer and *nmer* concentrations are given by $c_{x}=\frac{N_{x}}{A_{0}N_{\mathrm{grid}}}$. Thus, the unit grid area $A_{0}$ allows us to express the monomer and *nmer* concentrations predicted by reversible aggregation theory in terms of experimental units ($\mu m^{-2}$). We note that when $c_{\mathrm{mon}}=A_{0}^{-1},c_{\mathrm{nmer}}=A_{0}^{-1}$, then $\Delta G_{\begin{array}{c} mon\;to\\ nmer \end{array}}^{0}=0$. Thus, $A_{0}$ specifies the standard state concentration on the grid, serving the role of the 1.0 M standard state concentration convention in solution chemistry. With rearrangement of Eq. **1** we have

[2] $$\begin{array}{c} c_{\mathrm{nmer}}=A_{0}^{-1}{\left(A_{0}c_{mon}\right)}^{n}\;\text{exp}\left(\frac{-\Delta G_{\begin{array}{c} mon\;to\\ nmer \end{array}}^{0}}{RT}\right). \end{array}$$

To predict $c_{\mathrm{nmer}}$, we must know how $\Delta G_{\begin{array}{c} mon\;to\\ nmer \end{array}}^{0}$ in Eq. **2** changes as a function of n. To this end, we consider first the special case of a very large cluster on the grid (formally, an infinitely large cluster), which we call “bulk phase,” at equilibrium with monomers on the grid. This bulk phase is analogous to a solid or liquid condensate in equilibrium with a saturated solution. For the reaction mon⇌bulk phase, at equilibrium $G_{\mathrm{mon}}=G_{\mathrm{bulk}}$, where $G_{\mathrm{mon}}$ is defined above and $G_{\mathrm{bulk}}=G_{\mathrm{bulk}}^{0}$ for each bulk phase molecule under the assumption that the bulk phase is a uniform (and infinite) collection of indistinguishable proteins and thus does not contribute to the configurational entropy change when the reaction occurs. Therefore, for the reaction on the grid, $\begin{array}{c} \Delta G_{\begin{array}{c} mon\;to\\ bulk \end{array}}^{0}\equiv G_{\mathrm{bulk}}^{0}-G_{\mathrm{mon}}^{0}=RT\;\text{log}\left(\frac{N_{mon}}{N_{grid}}\right),\;N_{\mathrm{grid}}\gg N_{\mathrm{mon}} \end{array}$, and thus,

[3] $$c_{\begin{array}{c} mon\\ crit \end{array}}=A_{0}^{-1}\;\text{exp}\left(\frac{\Delta G_{\begin{array}{c} mon\;to\\ bulk \end{array}}^{0}}{RT}\right),$$

where we have applied $c_{x}=\frac{N_{x}}{A_{0}N_{\mathrm{grid}}}$ to the critical (saturated) monomer concentration $c_{\begin{array}{c} mon\\ crit \end{array}}$. Eq. **3** is a well-known expression for the critical monomer concentration at equilibrium with a bulk phase.

**Fig. 6.**
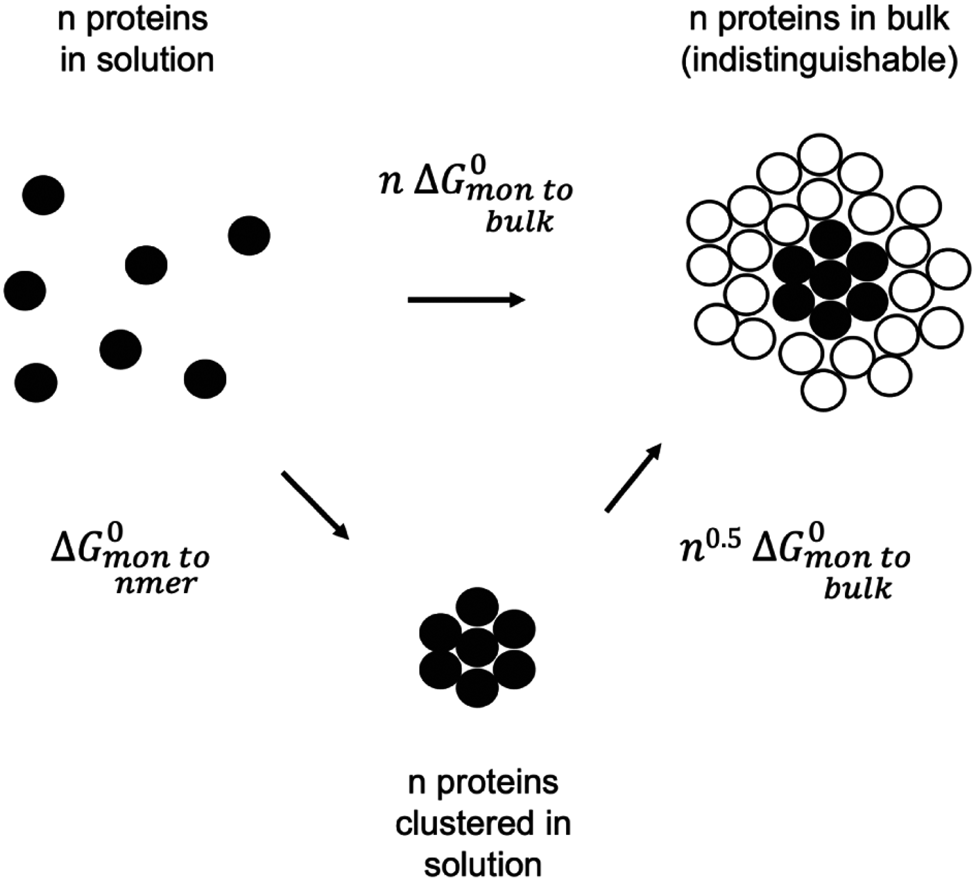
Thermodynamic cycle to determine $\Delta G_{\begin{array}{c} \mathrm{mon}\;to\\ nmer \end{array}}^{0}$ as a function of $\Delta G_{\begin{array}{c} \mathrm{mon}\;to\\ bulk \end{array}}^{0}$. The cartoon depicts two distinct paths for the transfer of n monomers to the bulk phase defined as a large sheet of closely packed proteins. The bottom path occurs in two steps with the formation of an intermediate cluster of n protein units. Black and white protein units aid visualization but are statistically indistinguishable.

Next consider the thought experiment outlined in Fig. 6. The thermodynamic cycle relates the transfer of n monomers from solution directly to the bulk phase (top) to a two-step process (bottom) in which an nmer is first formed, and then it is transferred to the bulk phase. The important point to note is that on average the proteins forming the nmer have incomplete neighbor bonding compared to an equal number of proteins embedded in the bulk phase, because some proteins are on the edge of the nmer. For two-dimensional nmers, the incomplete bonding is proportional to the number of proteins at the perimeter of the (compact) nmer and thus, for simplicity, we set it proportional to $n^{0.5}$, where we have noted that the nmer area is proportional to n (*SI Appendix*, Appendix 3). Fig. 6 expresses this idea using a proportionality constant of 1.0. Since at equilibrium the two paths in Fig. 6 for transferring *n* proteins in the membrane “solution” to the bulk phase must be equivalent,

[4] $$\Delta G_{\begin{array}{c} mon\;to\\ nmer \end{array}}^{0}=\left(n-n^{0.5}\right)\Delta G_{\begin{array}{c} mon\;to\\ bulk \end{array}}^{0}.$$

Eq. **4** expresses $\Delta G_{\begin{array}{c} mon\;to\\ nmer \end{array}}^{0}$ as a function of *n* for an idealized two-dimensional nmer. The term $n^{0.5}\Delta G_{\begin{array}{c} mon\;to\\ bulk \end{array}}^{0}$ is a boundary energy term subtracted from the standard free energy for transferring n monomers to the bulk phase ($n\Delta G_{\begin{array}{c} mon\;to\\ bulk \end{array}}^{0}$). The nmers observed in our experiments may have more complicated two-dimensional shapes and bonding properties (see below), however, proteins on their perimeters have fewer neighbor interactions, which is the origin of some form of boundary energy (*SI Appendix*, Appendix 3). Substitution of Eq. **4** into Eq. **2** yields an expression for the cluster size distribution for oligomerization of weakly interacting proteins according to the boundary energy in Fig. 6:

[5] $$\begin{array}{c} c_{\mathrm{nmer}}=A_{0}^{-1}(A_{0}c_{\mathrm{mon}}{)}^{n}\;\text{exp}\left[\frac{(n^{0.5}-n)\Delta G_{\begin{array}{c} mon\;to\\ bulk \end{array}}^{0}}{RT}\right]. \end{array}$$

Eq. **5** corresponds to dilute conditions where the approximations $N_{\mathrm{grid}}\gg N_{\mathrm{mon}}$, $N_{\mathrm{nmer}}$, are valid (*SI Appendix*, Appendix 2). The physical picture is that of clusters that are independent of, but in equilibrium with each other and with a bulk phase if it exists. The diagram in Fig. 6 depicts a bulk phase, but if the system has less than a critical monomer concentration, small clusters will still exist with a distribution given by Eq. **5**. The graph in Fig. 5*D* corresponds to Eq. **5** for a range of total protein concentrations, $c_{\mathrm{tot}}$. The predicted distributions always decrease monotonically, and notably, as $c_{\mathrm{tot}}$ is increased, a critical distribution is reached, and a bulk phase appears. This behavior is explicable as follows. When $c_{\mathrm{mon}}$ is given by Eq. **3**, that is, when $c_{\mathrm{mon}}=c_{\begin{array}{c} mon\\ crit \end{array}}$, Eq. **5** reduces to

[6] $$c_{\begin{array}{c} nmer\\ crit \end{array}}=A_{0}^{-1}\;\text{exp}\left(\frac{n^{0.5}\Delta G_{\begin{array}{c} mon\;to\\ bulk \end{array}}^{0}}{RT}\right).$$

At this critical concentration the total protein concentration contained within the distribution is,

[7] $$\begin{array}{c} c_{\mathrm{tot}}=A_{0}^{-1}\sum_{n=1}^{\infty }n\;\text{exp}\left(\frac{n^{0.5}\Delta G_{\begin{array}{c} mon\;to\\ bulk \end{array}}^{0}}{\mathrm{RT}}\right). \end{array}$$

The infinite series in Eq. **7** is convergent for negative $\Delta G_{\begin{array}{c} mon\;to\\ bulk \end{array}}^{0}$, which means $c_{\mathrm{tot}}$, the total number of proteins in the system, must be finite. This is a precise definition of the phase transition yielding a bulk phase out of two-dimensional clusters with a boundary energy $n^{0.5}\Delta G_{\begin{array}{c} mon\;to\\ bulk \end{array}}^{0}$. Other forms of the boundary energy will also produce a phase transition if Eq. **7** converges (*SI Appendix*, Appendix 3). The physical picture to have in mind is this: At total protein concentrations associated with $0< <c_{\mathrm{mon}}\leq A_{0}^{-1}\;\text{exp}\left(\frac{\Delta G_{\begin{array}{c} mon\;to\\ bulk \end{array}}^{0}}{RT}\right),$ self-clusters occur with a monotonically decreasing size distribution. We call the clusters that comprise this distribution higher-order transient structures (HOTS). For a total quantity of protein in the membrane greater than the sum in Eq. **7**, excess protein enters a bulk phase that will coexist with the critical HOTS distribution given by Eq. **6**.

The derivation of Eq. **5** contains several assumptions. In particular, the term $n^{0.5}\Delta G_{\begin{array}{c} mon\;to\\ bulk \end{array}}^{0}$ oversimplifies what is likely a more complex boundary energy. Regarding the shape of clusters in experiments, which seemingly are not compact (Fig. 2), we note that antibody labels have long extensions and gold labels are large, creating a false open appearance. By electron microscopy, when we look directly at membrane protein clusters, we observe that in fact they tend to be compact (*SI Appendix*, Appendix 6). Moreover, even though the observed clusters do not exactly match the idealized picture in Fig. 6, the essential aspect of nmer growth captured in the model is, when n monomers form an nmer there is an energy deficit (a boundary energy) compared to transferring the n monomers to a bulk phase. Depending on its mathematical form, this boundary energy may impose a limit on the total number of proteins that can exist in the HOTS distribution, causing it to reach a critical distribution and to initiate formation of a bulk phase (*SI Appendix*, Appendix 3).

**Fig. 7.**
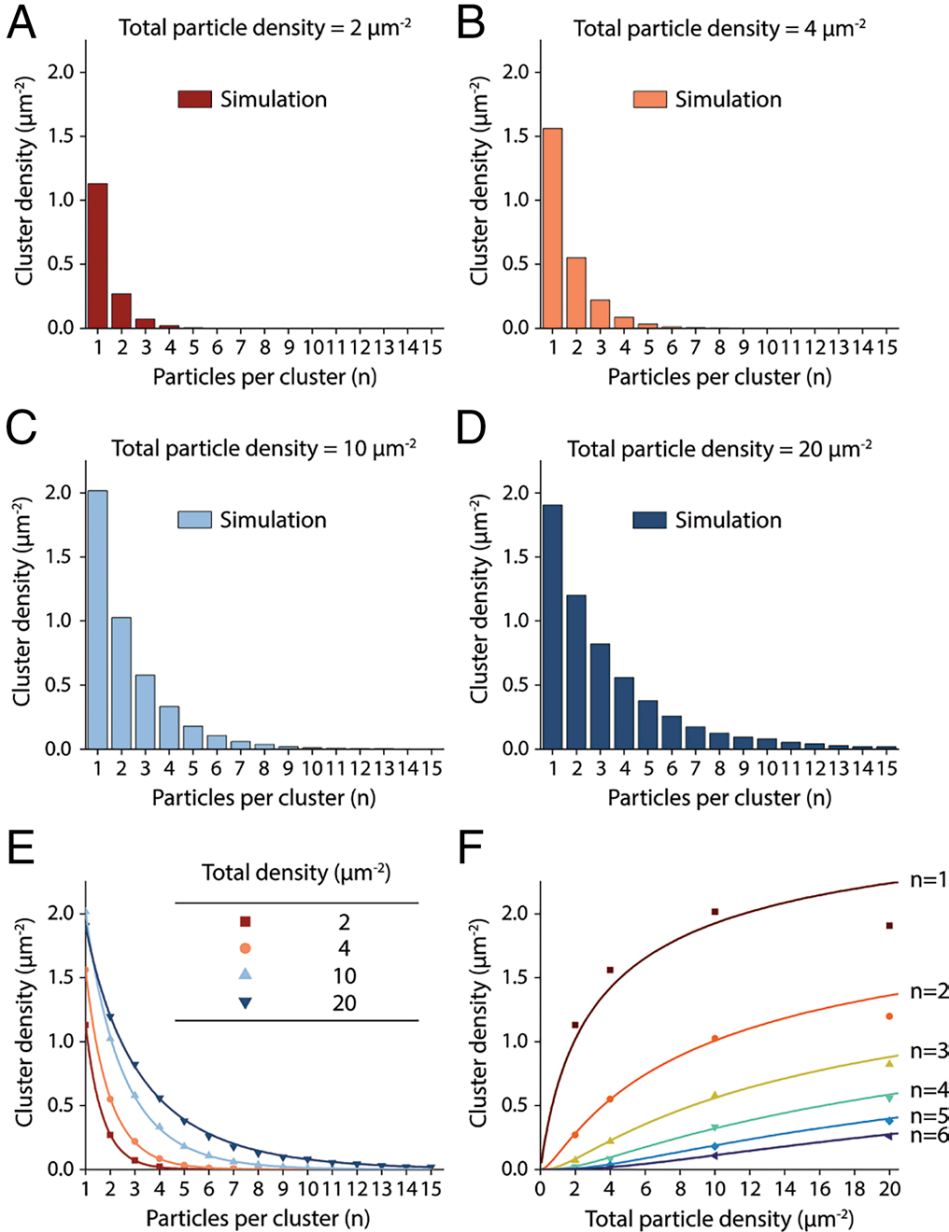
Simulated protein oligomers illustrate the self-assembly of membrane protein clusters through self-recognition. (*A*–*D*) Cluster size distributions of simulated diffusing “proteins” (particles) (*SI Appendix*, Movie S5) at the indicated total protein densities are shown. The proteins undergo a random walk on a square grid. If a protein has at least one neighbor, it will remain in place with a higher probability ($p_{\mathrm{wait}}=50$) than if it has no neighbor ($p_{\mathrm{wait}}=1$). See *Materials and Methods* and Mathematica code for details of the simulation and particle binning to generate histograms. (*E*) The cluster size distributions in panels (*A*−*D*) (symbols) are fit using Eq. **5** with Eq. **8** (curves) for the total protein densities in the legend. For all curves ${\Delta G}_{\begin{array}{c} mon\;to\\ bulk \end{array}}^{o}=-0.62\;RT$ and $A_{0}=0.15,$ 0.16, 0.18, 0.23 $\mu m^{2}$ for the protein densities $c_{\mathrm{tot}}=2,$ 4, 10, 20 $\mu m^{-2}$, respectively. (*F*) Cluster densities for *n* = 1 to 6 from the simulations (symbols) graphed as a function of total protein density ($c_{\mathrm{tot}}$). The curves correspond to Eq. **5** with $\Delta G_{\begin{array}{c} mon\;to\\ bulk \end{array}}^{o}=-0.62\;RT$ and $A_{0}=0.19\;{\text{μm}}^{2}$ (curves).

While the physical basis of the HOTS distribution can be explained by reversible aggregation theory, simulation of a simple “toy model” offers intuition on the process. This simulation serves to illustrate how membrane proteins can self-assemble into clusters through self-recognition and is not intended as a statistical thermodynamic description of HOTS. The movie (*SI Appendix*, Movie S5) shows 200 “proteins” undergoing a random walk on a 10,000-position square grid. In the beginning, with each iteration a protein either remains stationary or steps one position up, down, left, or right to an adjacent position if unoccupied: Each of these 5 outcomes occurs with equal probability. As the slide bar is moved, if a protein has at least one adjacent site occupied, the probability it will remain stationary increases, meaning its probability of dissociation, proportional to its off-rate, decreases. Notice that the presence of reversible binding in this simple simulation results in the occurrence of transient clusters mimicking HOTS. To visualize these, we note that the simulated clusters occupy multiple grid sites, not single grid sites as assumed above when deriving analytical expressions (*SI Appendix*, Appendix 2). Fig. 7 *A*–*D* shows cluster size distributions graphed from simulations like in *SI Appendix*, Movie S5 for different values of $c_{\mathrm{tot}}$, i.e., total protein concentration. At all concentrations $c_{\mathrm{tot}}$ the simulated size distributions are monotonically decreasing, and as $c_{\mathrm{tot}}$ is increased an asymptotic distribution is approached, in analogy to HOTS size distributions.

### Analyzing Experimental HOTS Size Distributions.

Experiments provide a measure of nmer concentration through counting proteins or labels and dividing by the measured membrane area. We fit Eq. **5** to these measured concentrations subject to the constraint that the sum of all terms in Eq. **5** equals $c_{\mathrm{tot}}$,

[8] $$c_{\mathrm{tot}}=\sum_{n=1}^{n_{\max}}n\;c_{\mathrm{nmer}},$$

where $n_{\max}$ denotes the largest measured n. Because $c_{\mathrm{mon}}$ and $c_{\mathrm{tot}}$ are measured, when fitting Eq. **5** constrained by Eq. **8**, determination of either one of $\Delta G_{\begin{array}{c} mon\;to\\ bulk \end{array}}^{0}$ or $A_{0}$ specifies the other. In other words, the shape of the experimental HOTS size distribution, interpreted through the model of the boundary energy, $n^{0.5}\Delta G_{\begin{array}{c} mon\;to\\ bulk \end{array}}^{0}$, estimates values for both $\Delta G_{\begin{array}{c} mon\;to\\ bulk \end{array}}^{0}$ and $A_{0}$, the latter setting the standard state concentration, $A_{0}^{-1}$. A more detailed development of reversible aggregation theory shows that for a given (measured) $c_{\mathrm{mon}}$ the value of $A_{0}$ must be adjusted to fix $c_{\mathrm{tot}}$ (*SI Appendix*, Appendix 3).

In *SI Appendix*, Appendix 4 we explore the dependence of $c_{\mathrm{nmer}}$ in Eq. **5** with the constraint in Eq. **8** on the values of $A_{0}$ and $\Delta G_{\begin{array}{c} mon\;to\\ bulk \end{array}}^{0}$. We find that, at a given (small) $c_{\mathrm{tot}}$, a large enough $A_{0}$ allows for broad HOTS size distributions even at small magnitudes of $\Delta G_{\begin{array}{c} mon\;to\\ bulk \end{array}}^{0}$. Thus, large $A_{0}$ permit appreciable HOTS concentrations for weakly interacting proteins at small $c_{\mathrm{tot}}$. While recognizing that our analysis depends on the specific model of cluster formation described in Fig. 6, we note that the data distinguish $A_{0}$ and $\Delta G_{\begin{array}{c} mon\;to\\ bulk \end{array}}^{0}$ because they enter Eq. **5** through distinct powers of n. Because $A_{0}$ and $\Delta G_{\begin{array}{c} mon\;to\\ bulk \end{array}}^{0}$ derived from fitting data depend on unknown details of the self-assembly process, we do not interpret their precise numerical values. However, it is useful to keep in mind that $\Delta G_{\begin{array}{c} mon\;to\\ bulk \end{array}}^{0}$ is related to the cohesive strength of protein binding and $A_{0}$ is related to the configurational entropy of the oligomerization reaction.

One could reinterpret Eq. **5** with Eq. **8** to introduce an arbitrary unit grid area, $A_{0_{\mathrm{ref}}}$, such that $A_{0}=\gamma A_{0_{\mathrm{ref}}}$ in Eq. **5**. When this form of the distribution equation is fit to experimental data with the constraint in Eq. **8** (containing $A_{0}=\gamma A_{0_{\mathrm{ref}}}$) one obtains estimates for $\Delta G_{\begin{array}{c} mon\;to\\ bulk \end{array}}^{0}$ and γ, rather than $\Delta G_{\begin{array}{c} mon\;to\\ bulk \end{array}}^{0}$ and $A_{0}$. In this case, γ could be interpreted as a multiplier to the experimental monomer and *nmer* concentrations, the product being the thermodynamic activity. This would be an equivalent approach. Here, for interpretive reasons given below, we focus on the value of $A_{0}$ as the observed unit (grid) area that comes directly from the fitting procedure, and we call $\sqrt{A_{0}}$ the configurational length scale of the system.

To gain intuition on how to compare our theoretical predictions to our experimental results, it is instructive to first fit the theory in Eq. **5** with Eq. **8** to the cluster size distributions generated in the toy model simulations (Fig. 7). All fitted curves in Fig. 7*E* correspond to a single global value of $\Delta G_{\begin{array}{c} mon\;to\\ bulk \end{array}}^{0}$ in Eq. **5**, with $A_{0}$ determined for individual curves by the value of $c_{\mathrm{tot}}$ through Eq. **8** (Fig. 7*E*). We can then directly compare the unit grid area used in the simulations ($0.01\;\mu m^{2}$) to the values of $A_{0}$ obtained from the fits to the theory. These begin at $0.15\;\mu m^{2}$ at the lowest protein concentration and increase to $0.23\;\mu m^{2}$ at the highest concentration. The disagreement between the grid area used in the simulations and the values of $A_{0}$ obtained from the fits at first seems perplexing, but there are several reasons for the difference. First, protein binding is treated differently in the simulations and in Eq. **5** with Eq. **8**. In particular, the simulation used for Fig. 7 is not formulated in terms of an *nmer* energy. Second, the simulation is run on a small grid (10^4^ positions), so the approximation of infinite dilution (i.e., $N_{\mathrm{grid}}\gg N_{mon},N_{nmer}$) is not met. And third, the simulated clusters occur on multiple grid sites instead of being treated as point particles, as assumed in Eq. **5** with Eq. **8**. In other words, the analytical expressions are for a specific model of the HOTS energy as described in Fig. 6, and an ideal system, while the simulations use different approximations. These differences notwithstanding, the simulated cluster size distributions exhibit the general features of Eq. **5** with Eq. **8**.

**Fig. 8.**
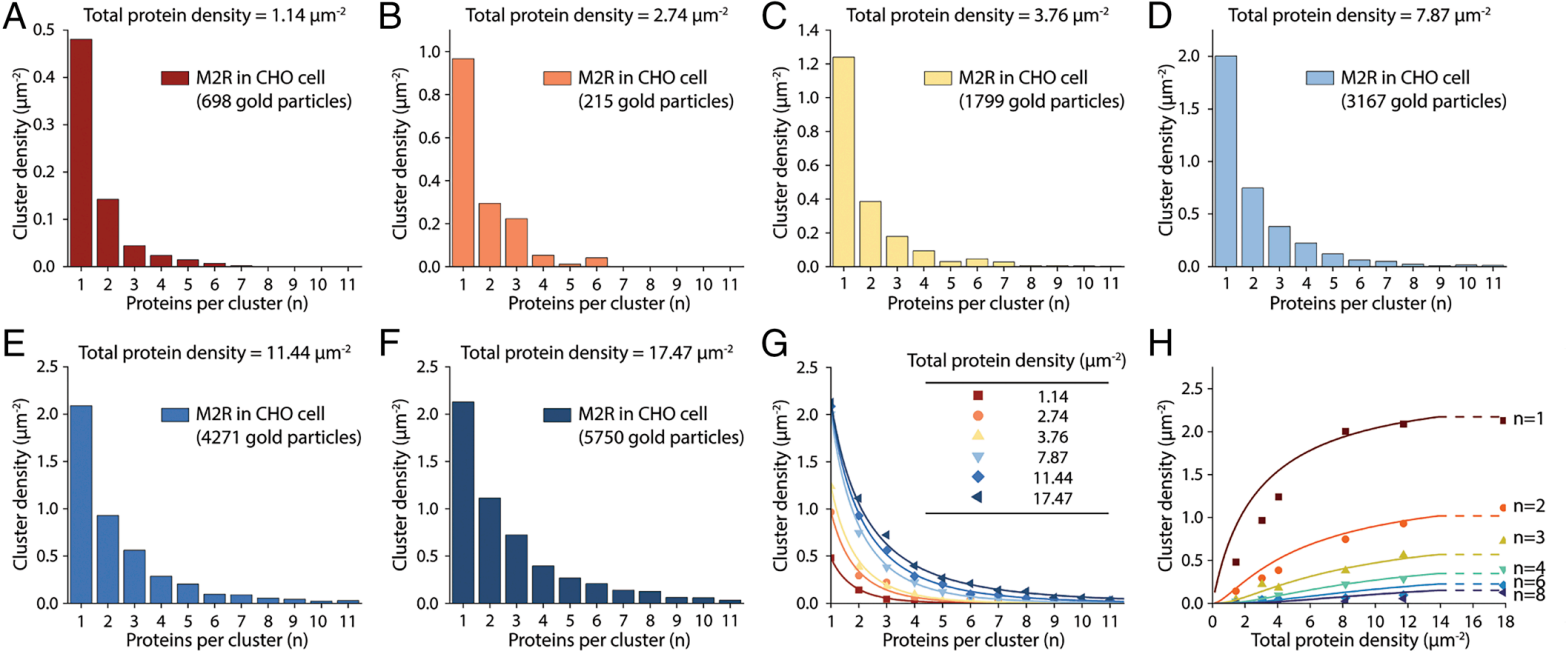
M2R cluster size distributions in CHO cells correspond to a HOTS self-assembly model. (*A*−*F*) M2R cluster size distributions in CHO cells expressed to the indicated total protein densities are shown. Proteins were labeled with 18 nm gold particles. Nonspecific gold particle labeling was estimated using CHO cells without heterologous expression and subtracted. Each panel represents the result of one unroofed membrane (one cell). The total numbers of gold particles measured and analyzed are indicated. (*G*) Cluster size distributions from panels (*A*−*F*) are shown (symbols). The solid curves correspond to a fit to Eq. **5** constrained by Eq. **8** using the measured total protein densities listed (inset). For all curves $\Delta G_{\begin{array}{c} mon\;to\\ bulk \end{array}}^{0}=-1.82\;RT$ and $A_{0}=0.22,$ 0.13, 0.10, 0.07, 0.08, 0.08 μm^2^ for the lowest to the highest protein concentrations, respectively. (*H*) Cluster densities for *n* = 1 to 8 from data shown in (*A*−*F*) graphed as a function of measured total protein density ($c_{\mathrm{tot}}$). The curves correspond to Eq. **5** with $\Delta G_{\begin{array}{c} mon\;to\\ bulk \end{array}}^{0}=-1.82\;RT$ and $A_{0}=0.08\;{\mathrm{\mu m}}^{2}$.

Next, we examine experimental cluster size distributions for M2R in the context of our simplified model (Fig. 8). Because HL-1 cells express a constant M2R concentration, to vary $c_{\mathrm{tot}}$ we used heterologous expression in CHO cells. Cluster size distributions are shown for six M2R concentrations (Fig. 8 *A*−*F*). The fitted curves (Fig. 8 *G* and *H*) correspond to a single global value of $\Delta G_{\begin{array}{c} mon\;to\\ bulk \end{array}}^{0}$ with $A_{0}$ determined for individual curves by the value of $c_{\mathrm{tot}}$ through Eq. **8**. $A_{0}$ remains nearly constant over all curves, excepting the lowest concentration where the data statistics are poorer owing to smaller numbers. The general behavior of the measured M2R cluster size distributions is consistent with a HOTS size distribution as defined by our reversible aggregation theory. Likewise, when we express β1AR, GIRK channels, A1R, and AC at different levels in CHO cells and label them as we have described, they all exhibit qualitatively similar behavior (*SI Appendix*, Fig. S6).

**Fig. 9.**
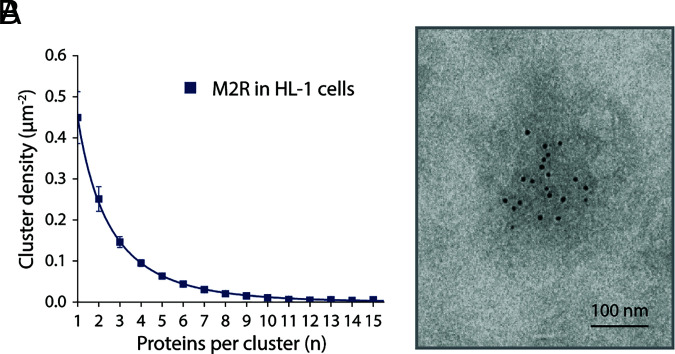
The HOTS size distribution of M2R in HL-1 cells. (*A*) The cluster size distribution of M2Rs labeled with 6 nm gold particles in HL-1 cells (symbols) is fit to Eq. **5** constrained by Eq. **8**, yielding $\Delta G_{\begin{array}{c} mon\;to\\ bulk \end{array}}^{0}=-1.24\;RT$ and $A_{0}=0.58\;{\text{μm}}^{2}$ (curve). Nonspecific gold particle labeling was estimated from CHO cells without heterologous expression and subtracted. Data represent mean and SE from 13 electron microscope montages. (*B*) Representative negative stain electron micrograph of a larger M2R cluster in HL-1 cells. Proteins are labeled with 6 nm gold particles. (Scale bar, 100 nm.)

While HL-1 cells naturally express an approximately constant total concentration of M2R, yielding the HOTS size distribution shown (Fig. 3*A*), we ask, where is this distribution relative to the predicted critical distribution? Fig. 9*A* shows M2R data for HL-1 cells (from Fig. 3*A*) with a curve corresponding to Eq. **5** with Eq. **8** using $\Delta G_{\begin{array}{c} mon\;to\\ bulk \end{array}}^{0}=-1.24\;RT$ and $A_{0}=0.58\;\mu m^{2}$. Substitution of these values into Eq. **3** yields $c_{\begin{array}{c} mon\\ crit \end{array}}=0.50\;\mu m^{-2}$. We see from Fig. 3*A* that $c_{\mathrm{mon}}=0.45\;\mu m^{-2}$. Thus, the HOTS size distribution in HL-1 cells is close ($\frac{c_{mon}}{c_{\begin{array}{c} mon\\ crit \end{array}}}=0.90$) to the critical distribution predicted by reversible aggregation theory. Note that Eq. **5**, with $\Delta G_{\begin{array}{c} mon\;to\\ bulk \end{array}}^{0}=-1.24\;RT$ and $A_{0}=0.58\;\mu m^{2}$, gives an expectation of 345 proteins (out of 12,773 total) over the cluster size range n = 20 to n = 29. But we observe 929 proteins over this range. Thus, Eq. **5** yields the observed distribution of small HOTS (Fig. 9*A*), but the observed larger clusters exceed theoretical predictions. Recall that Eq. **5** accounts for proteins in the distribution of HOTS up to their critical concentration. We suggest that excess large clusters, like the one shown in Fig. 9*B*, reflect the occurrence of outlier bulk phase clusters, which are expected to self-assemble if M2R reaches and goes slightly beyond its critical concentration in the membrane of HL-1 cells.

A technical concern with quantification of HOTS size distributions using gold labels is that we undoubtedly underlabel proteins to some extent. We find, for example, that different gold particles label with variable efficiencies, giving receptor densities that can vary by approximately two-fold. And yet the data analysis yields qualitatively similar results. *SI Appendix*, Appendix 5 provides an explanation of robustness of cluster size distributions to modest variations in labeling efficiency.

Owing to length limitations, two sections of Results have been moved to *SI Appendix*, Appendix 6. These results are summarized here.

**HOTS and bulk phase cluster formation through oligomerization in reconstituted membranes.** This section shows 1) that HOTS form spontaneously in a reconstituted system containing a single protein species, 2) that proteins inside HOTS are sufficiently close to touch each other, and 3) that the occurrence and duration of dimerization events between individual M2Rs can be measured in a synthetic freestanding membrane.

**The dynamical nature of HOTS.** This section shows that oligomerization of weakly interacting proteins in cells is a dynamic process and HOTS, as their name indicates, are transient objects that rapidly exchange their protein components with each other.

## Discussion

### Summary of Data Supporting the HOTS Hypothesis.

Our key experimental observations are the following: 1) Five plasma membrane proteins form small self-clusters at native concentrations in HL-1 cells. 2) The cluster size distributions are monotonically decreasing. 3) For M2R expressed at different concentrations in CHO cells, an asymptotic cluster size distribution is approached as the total protein concentration is increased. 4) Spontaneous cluster formation occurs in reconstituted lipid membranes and proteins interact with each other in diffusion experiments. At sufficiently high protein concentrations a monotonically decreasing size distribution of small clusters coexists with large, bulk phase clusters. 5) Clusters in the plasma membrane are associated with the rapid exchange of constituent protein units.

The first observation of separate clusters for each protein type would be consistent with the cell making a specific attractor, a scaffold, or raft, for each protein type. But self-attraction is a simpler explanation, i.e., requiring fewer components, and is consistent with the monotonically decreasing cluster size distribution and its asymptotic behavior as the total protein concentration is increased. We considered whether type-specific clusters might reflect synthesis and insertion of multiple copies of like proteins, which then remain together. But this “born together stay together” principle is inconsistent with the observation that clusters rapidly exchange their constituent protein units in the plasma membrane. Finally, the occurrence of clusters and the reversible binding of diffusing proteins in a reconstituted system without other components strongly support a self-assembly mechanism.

Self-assembly under many (indeed most) circumstances does not yield a monotonically decreasing cluster size distribution. In contrast, the HOTS distributions described here are monotonically decreasing, and approach an asymptotic, monotonically decreasing distribution as a critical concentration is approached. HOTS occur both at a subcritical concentration, and at a critical concentration when in equilibrium with a bulk phase.

### Alignment of Published Data with the HOTS Hypothesis.

The average HOTS size is so small that common superresolution fluorescence microscopy methods do not easily distinguish between monomers and small clusters, because of rapid photobleaching and insufficient spatial resolution. This is why they have been difficult to identify and characterize quantitatively. However, there are ample independent data consistent with their existence. Single-molecule tracking studies show that class A GPCRs, which are functional as monomers, and other membrane proteins, dimerize and sometimes form higher-order oligomers (44–46). In addition, experiments employing resonance energy transfer (47), photobleaching (48, 49), immunogold labeling (50–52), deduction from electrophysiological recordings (53), and atomic structures (54) also support oligomerization of various membrane proteins (12). These other studies did not quantify the size distribution of oligomers, but they clearly showed that oligomerization occurs with many membrane proteins.

In experiments using freeze fracture and immunogold labeling of mouse Purkinje neurons, GABA_B_ receptors were shown to exhibit a monotonically decreasing cluster size distribution (52). While the basis for the distribution was not addressed in the study, it appears consistent with a HOTS distribution. Experiments using superresolution optical microscopy to estimate cluster area have also uncovered monotonically decreasing size distributions (53, 55, 56). In one study the distribution was fit using a kinetic model that invoked rates of nucleation, growth, and removal (55). This kinetic model, called “stochastic self-assembly,” is very different from the framework considered here because the model of cluster formation is irreversible except by removal (recycling or degradation) of the clusters.

Many studies have relied on heterologous expression of membrane proteins. In some, very large (micrometer-sized) clusters like those in *SI Appendix*, Movie S6 are seen. These are consistent with bulk phase clusters. But as we described above, the bulk phase clusters should coexist with small HOTS, which in principle should be like those present at native expression levels. We think that the HOTS hypothesis is consistent with many published results in the fields of GPCR signaling, single particle tracking, and ion channel physiology.

### The Relationship Between HOTS and Bulk Phase Clusters.

The HOTS distribution is the outcome of an equilibrium balance between the opposing contributions of configurational entropy, which favors monomers, and the attractive interactions between proteins, which favors oligomers. In other words, this is the “natural” equilibrium distribution for oligomerization in the subcritical regime, where configurational entropy is central to its form.

The occurrence of a phase transition does not mean that the size distribution of HOTS includes an abundance of large clusters. Instead, it means the following. At protein concentrations below a critical level, as more proteins are added to the membrane, the concentration of monomers and HOTS grows asymptotically with the size distribution broadening but maintaining its monotonically decreasing form, as shown in Fig. 5*D*. The chemical potential of each protein unit in the distribution of HOTS also increases until eventually it equals that in a continuous sheet of the proteins, a bulk phase. At this point, a critical distribution of HOTS exists; proteins exceeding the number in the critical distribution go to the bulk phase, just as solute molecules added to a saturated solution go to a separate solid or liquid phase. Therefore, large bulk phase clusters do not “belong” to the distribution of HOTS, but they are in equilibrium with it. In other words, small HOTS and large bulk phase clusters are distinct, but they are both manifestations of the same thermodynamic process and therefore must originate from the same cohesive interactions between the proteins.

Whether a phase transition can occur is in general related to the *n*-dependence of the HOTS boundary energy, expressed as $n^{0.5}\Delta G_{\begin{array}{c} mon\;to\\ bulk \end{array}}^{0}$ for our simplified model of two-dimensional clusters (*SI Appendix*, Appendix 3). This means that critical behavior is fundamentally related to the number of ways that a protein can interact with its neighbors. Linear clusters, i.e., polymer chains, do not give rise to a phase transition because the unbonded ends of a linear chain typically remain the same as the chain grows (43). Where (i.e., at what concentration) a phase transition occurs is expressed by Eq. **3**. For a given value of $A_{0}$, if the protein units interact strongly (large negative $\Delta G_{\begin{array}{c} mon\;to\\ bulk \end{array}}^{0}$), the phase transition occurs at a low protein concentration. Thus, strongly interacting proteins, unless very dilute, will exist mainly as bulk phase clusters. Small nmer structures will still coexist with the bulk phase even in this case, but at very low concentrations, with the monomer concentration equal to a very small $c_{\begin{array}{c} mon\\ crit \end{array}}$ (Eq. **3**) and dimers, trimers, etc. at progressively decreasing concentrations (Eq. **6**).

Our data suggest that the membrane proteins we studied in HL-1 cells exist mainly as small HOTS. But many examples of large sheets of one kind of protein (i.e., a bulk phase) occurring naturally are well known, e.g., rhodopsin molecules in photoreceptor membranes (57). We note that a lipid membrane itself is a two-dimensional sheet of bulk phase lipid molecules. For proteins in the membrane, there is likely a continuum of interaction energies from weak to strong. Small, transient HOTS of certain membrane proteins may fulfill one purpose, for example, rapid on–off signaling, whereas larger, more permanent clusters of other membrane proteins could mediate longer-lived processes. One might also imagine that the binding of a ligand or a phosphorylation event could induce or diminish clustering through a sudden change in the value of its $\Delta G_{\begin{array}{c} mon\;to\\ bulk \end{array}}^{0}$ (45, 56, 58).

### Weakly Interacting Proteins and the Configurational Length Scale.

If interactions between proteins are weak, a sufficiently high membrane protein concentration should be required to force oligomerization by mass action. But HOTS are observed in HL-1 cells at a total M2R concentration of just a few per μm^2^. Interpreted in the context of our reversible aggregation theory, this is because $A_{0}$ is large, ~0.58 μm^2^ for M2R in HL-1 cells (*SI Appendix*, Appendix 4). Recall that $A_{0}$ can be viewed as the unit area in the grid model, used to calculate the configurational entropy term (*SI Appendix*, Appendix 2). Considering that a disk with a cross-sectional area consistent with the M2R area (~20 nm^2^) can adopt about 30,000 unique, nonoverlapping positions in an area 0.58 μm^2^, we conclude that $A_{0}$ is absurdly large. To restate, the data interpreted through the grid model imply that the cell membrane somehow vastly reduces the possible configurations (manifest in our model as a coarse grid spacing) that we would intuitively think a membrane protein should be able to sample. Consequently, the membrane proteins behave as if they are much more concentrated than they really are, so that oligomers are formed even though interactions between them are weak. It is known that oligomerization reactions occurring in the cytoplasm are shifted toward oligomerization compared to dilute solutions owing to molecular crowding (59, 60). We propose that the large $A_{0}$ found here reflects, at least in part, a crowded membrane environment filled with many different proteins that limit accessible configurations. A prediction of this hypothesis is that a protein reconstituted into pure lipid bilayers, in the absence of other crowding proteins, should require higher concentrations to form HOTS and bulk phase clusters. Indeed, we find in reconstitution experiments (see *SI Appendix*, Appendix 6) that proteins must be present at concentrations 10^2^ to 10^3^ times higher than in cell membranes to show similar levels of oligomerization.

It could be that compartments of living systems, including membranes, have physical properties that determine the local configurational length scale, $\sqrt{A_{0}}$. Through regulation of those physical properties, a cell could control oligomerization reactions by adjusting the configurational entropy term in the reaction free energy. This effect, we propose, permits HOTS to occur in proteins that interact weakly with themselves, even though they are present in the membrane at relatively low concentrations. We noted earlier that the HOTS observed in cell membranes coincide with protein-rich (protein island) membrane regions (i.e., patches with dark backgrounds on negative stain EM images). We hypothesize this is so because protein-rich regions modify the configurational length scale through molecular crowding.

### The Molecular Origins of Self-Oligomerization.

An important feature of the model presented here is that proteins exhibit self-attraction with specificity. Specificity, or molecular recognition, is required to explain why M2R oligomerizes with M2R, β1AR with β1AR, etc. We found that clustered M2Rs in HL-1 cells are close enough to contact their neighbors directly (Fig. 4*E*). Also, when determining structures of ion channels reconstituted into lipid vesicles (61) and giant plasma membrane vesicles (62), we have observed that channels in clusters are always close enough to make direct contacts through elements of their structure. Atomic structures of the CXCR4 receptor in various oligomeric configurations demonstrate how a GPCR can recognize and bind to itself through multiple surfaces (54). Mutational (56, 63), FRET (47), and BRET (48) studies also point to protein–protein interactions as a basis for membrane protein oligomerization. Thus, it is easy to understand how proteins can recognize themselves through matched interfaces. In addition to protein–protein and protein–peptide interactions that would account for traditional molecular recognition, influences of protein shape mediated locally through the membrane, or specific lipid molecules that might assist these interactions, could also play a role (16, 64, 65).

Zooming out from the mechanistic details, it occurs to us that if it is true that many membrane proteins form HOTS because they have evolved surfaces to self-oligomerize, then the process would of course be genetically encoded. The name higher-order transient structure reflects the idea that HOTS are a kind of supramolecular unit. If HOTS are genetically selected entities, they ought to be beneficial in some way. In the accompanying paper, we present evidence that HOTS facilitate M2R regulation of GIRK channels in cell membranes (19).

## Materials and Methods Summary (*SI Appendix*, Supporting Information).

### Protein Expression.

Proteins in HL-1 cells were expressed at endogenous levels. Proteins in CHO cells were overexpressed by virus infection. Protein for reconstitution was expressed in HEK293S GnTI^−^ cells by virus infection.

### Detection of Protein Distributions.

Single proteins were identified using immunogold labels with an electron microscope and for M2R using a fluorescent antagonist with cryogenic light microscopy.

### Physiology.

Electrophysiological recordings and spontaneous Ca^2+^ oscillations were measured in HL-1 cells using whole-cell patch recording and fluorescence measurements with a Ca^2+^ indicator.

### Reconstitution.

Proteins were purified and reconstituted for analysis by electron microscopy or by fluorescence microscopy after fusion into freestanding bilayers.

### Simulations and Calculations.

Were carried out using Mathematica.

## Supplementary Material

Appendix 01 (PDF)

Movie S1. Representative movies of spontaneous calcium oscillations in confluent HL-1 cells. Confluent HL-1 cells are loaded with Fluo-8 AM and imaged. Spontaneous calcium oscillations were observed at basal level (Movie S1), accelerated by isoproterenol (Movie S2) and slowed by carbachol (Movie S3) and adenosine (Movie S4). 10 μM isoproterenol, 10 μM carbachol, and 10 μM adenosine were used.

Movie S2. Representative movies of spontaneous calcium oscillations in confluent HL-1 cells. Confluent HL-1 cells are loaded with Fluo-8 AM and imaged. Spontaneous calcium oscillations were observed at basal level (Movie S1), accelerated by isoproterenol (Movie S2) and slowed by carbachol (Movie S3) and adenosine (Movie S4). 10 μM isoproterenol, 10 μM carbachol, and 10 μM adenosine were used.

Movie S3. Representative movies of spontaneous calcium oscillations in confluent HL-1 cells. Confluent HL-1 cells are loaded with Fluo-8 AM and imaged. Spontaneous calcium oscillations were observed at basal level (Movie S1), accelerated by isoproterenol (Movie S2) and slowed by carbachol (Movie S3) and adenosine (Movie S4). 10 μM isoproterenol, 10 μM carbachol, and 10 μM adenosine were used.

Movie S4. Representative movies of spontaneous calcium oscillations in confluent HL-1 cells. Confluent HL-1 cells are loaded with Fluo-8 AM and imaged. Spontaneous calcium oscillations were observed at basal level (Movie S1), accelerated by isoproterenol (Movie S2) and slowed by carbachol (Movie S3) and adenosine (Movie S4). 10 μM isoproterenol, 10 μM carbachol, and 10 μM adenosine were used.

Movie S5. Diffusion of interacting particles on a grid. 200 particles undergo a random walk on a 100 × 100 (10,000 site) 2-dimensional grid such that two particles cannot occupy the same site. As the slide bar is moved, if a particle has at least one neighbor, the probability it will not step to a neighboring site (if unoccupied by a particle) is increased by a constant weight. The dimensional edge length is assigned through real distance per integer unit of grid length (*rpg*) and time per frame by (rpg)^2^/4*D*, where *D* is the diffusion coefficient. Code is available at (MacKinnon Lab website). We emphasize that the simulations considered here are not based on an nmer energy function (see Appendix 3), and are not set up to examine the equilibrium (Boltzmann) HOTS distribution (24). The toy model serves to illustrate, in very simple terms, how particles can self-assemble into clusters through self-recognition.

Movie S6. Representative movie of 40 nm gold-labeled HA-M2R in PtK2 cells. HA-M2Rs were labeled with a fluorescent antibody, then sparsely labeled with a secondary antibody containing a single 40 nm gold particle and imaged under differential interference contrast microscopy. The video was acquired at 6.6 frames/s and lasted 10 min. Fluorescent images were taken before and after the video was recorded to ensure that large puncta remained relatively stationary. The gold movie was superimposed on a fluorescent image of the same region of the membrane. The movie is accelerated four times.

## Data Availability

All study data are included in the article and/or supporting information.
