# Supplementary material for: Higher-order transient membrane protein structures
Source: Proc Natl Acad Sci U S A. 2024 Dec 31;122(1):e2421275121. doi: 10.1073/pnas.2421275121 (PMC11725870; doi:10.1073/pnas.2421275121)
Supplement: Supplementary file 1 — Appendix 01 (PDF) [file pnas.2421275121.sapp.pdf]

PNAS

Supporting Information for
Higher-order transient membrane protein structures

Yuxi Zhang[1,2], Hisham Mazal[3,4], Venkata Shiva Mandala[1,2], Gonzalo Pérez-Mitta[1,2], Vahid Sondoghdar[3,4,5], Christoph A. Haselwandter[6,7], and Roderick MacKinnon[1,2]

[1]Laboratory of Molecular Neurobiology and Biophysics, The Rockefeller University, New York, NY 10065. [2]Howard Hughes Medical Institute, The Rockefeller University, New York, NY 10065. [3]Max Planck Institute for the Science of Light, Erlangen 91058, Germany. [4]Max-Planck-Zentrum für Physik und Medizin, 91058 Erlangen, Germany. [5]Department of Physics, Friedrich Alexander University Erlangen-Nürnberg, Erlangen 91058, Germany. [6]Department of Physics and Astronomy, University of Southern California, Los Angeles, CA 90089. [7]Department of Quantitative and Computational Biology, University of Southern California, Los Angeles, CA 90089

Roderick MacKinnon
Email: mackinn@rockefeller.edu
Christoph A. Haselwandter
Email: cah77@usc.edu

This PDF file includes:

Appendices 1 to 6
Figures S1 to S7
SI Materials and Methods
Legends for Movies S1 to S6
SI References

Other supporting materials for this manuscript include the following:

Movies S1 to S6

Appendix 1: Attractor model by equilibrium association

This appendix presents the cluster size distribution for a model in which membrane proteins diffusing in the membrane are attracted to regions of the membrane where they can bind reversibly. Here, the attractors are assumed to be uniform in size, randomly distributed, and unlimited in binding capacity. In this model, proteins associate to and dissociate from an attractor with rate constants k_{on} and k_{off}, respectively. The system is taken to be at equilibrium (all concentrations stationary and no net flux between species). For the association of a protein at concentration $[x]$ with an attractor at concentration $[m]$ to form a complex at concentration $[x_1 m]$ the equilibrium association constant K_a is

$$K_a \equiv \frac{k_{on}}{k_{off}} = \frac{[x_1 m]}{[x]\,[m]} \,. \qquad \text{Eq. S1}$$

Likewise, for the reaction that adds an ith protein to an attractor with $i-1$ proteins already present,

$$K_i = \frac{[x_i m]}{[x]\,[x_{i-1} m]} \,. \qquad \text{Eq. S2}$$

Through recursion, we express the concentration of attractors containing n proteins as a function of $[x]$, $[m]$, and K_i,

$$[x_n m] = [x]^n\,[m] \prod_{i=1}^{n} K_i \,, \qquad \text{Eq. S3}$$

where we use the notation $[x_0 m] \equiv [m]$. By the following statistical reasoning, K_i can be expressed as a function of K_a: The association rate for the ith addition of a protein to an attractor is presumed independent of attractor occupancy (because binding is unlimited), whereas the dissociation rate should be proportional to the number of proteins in the attractor (because the probability of dissociation increases in proportion to the number of proteins in the attractor), thus, $K_i = \frac{k_{on}}{i\,k_{off}} = \frac{K_a}{i}$. Substitution of this result into Eq. S3 yields

$$[x_n m] = \frac{{K_a}^n}{n!}[x]^n\,[m] \,. \qquad \text{Eq. S4}$$

The total concentration of protein in the membrane is given by

$$ctot = [x] + \sum_{n=1}^{\infty} n\,[x_n m] = [x] + K_a\,[x]\,[m]\,e^{K_a\,[x]} \,, \qquad \text{Eq. S5}$$

and the total concentration of attractors in the membrane is given by

$$mtot = \sum_{n=0}^{\infty} [x_n m] = [m]\,e^{K_a\,[x]} \,. \qquad \text{Eq. S6}$$

Solving Eqs. S5 and S6 for $[x]$ and $[m]$ as a function of $ctot$ and $mtot$ and substituting the results into Eq. S4 we find

$$[x_n m] = \frac{1}{n!}\,{K_a}^n\,mtot\,e^{-\frac{K_a\,ctot}{1+K_a\,mtot}} \left(\frac{ctot}{1+K_a\,mtot}\right)^n \,. \qquad \text{Eq. S7}$$

The number of proteins inside attractors is given by

$$\sum_{n=1}^{\infty} n\,[x_n m] = \frac{K_a\, ctot\, mtot}{1 + K_a\, mtot}\ , \qquad \text{Eq. S8}$$

and therefore, because proteins outside attractors are assumed to be monomers, the cluster size distribution of proteins in the membrane (inside and outside attractors) is given by

$$f(n) = \frac{1}{n!}\, {K_a}^n\, mtot\, e^{-\frac{K_a\, ctot}{1+K_a\, mtot}} \left(\frac{ctot}{1 + K_a\, mtot}\right)^n + \left(ctot - \frac{K_a\, ctot\, mtot}{1 + K_a\, mtot}\right) \delta_{n,1}\ , \qquad \text{Eq. S9}$$

where $\delta_{i,j}$ is the Kronecker delta. The curves in Figure 5B in the main text are obtained from Eq. S9 with the values of K_a, $mtot$, and $ctot$ provided in this figure and its caption.

Appendix 2: Self-assembly model: Derivation of the HOTS distribution on a grid

As described in the main text, the idealized system considered here is a planar surface represented as a 2-dimensional grid with N_{grid} positions on which proteins undergo a random walk. The grid has a fixed area equal to $A_0\, N_{grid}$, where A_0 is the unit grid area. When proteins enter the same grid position, they can bind reversibly to form an *nmer*. We assume here that an *nmer* also occupies one grid position, and that the *nmers* also undergo a random walk. We consider the oligomerization reaction between *n* monomers and one *nmer*, $n\, mon \rightleftharpoons 1\, nmer$, in thermal equilibrium with a heat bath at constant temperature and pressure. Under the assumption that the system is at equilibrium, the second law of thermodynamics mandates that the total entropy change associated with the reaction be zero (1):

$$\Delta S_{tot} \equiv \Delta S_{th} + \Delta S_{surr} + \Delta S_{\substack{cf\ mon \\ to\ nmer}} = 0\,, \qquad \text{Eq. S10}$$

where ΔS_{th} is the difference between the thermal entropies of the pure products and pure reactants, ΔS_{surr} is the change in entropy of the surrounding heat bath, $\frac{-\Delta H}{T}$, ΔH being the change in enthalpy, and ΔS_{cf} is the change in the configurational entropy when *n* monomers form one *nmer*. To calculate ΔS_{cf} within the grid model we compute the number of microstates, Ω, before and after the reaction,

$$\Omega_{initial} = \frac{N_{grid}!}{N_{mon}!\, N_{nmer}!\, (N_{grid} - N_{mon} - N_{nmer})!}\,,$$

$$\Omega_{final} = \frac{N_{grid}!}{(N_{mon} - n)!\, (N_{nmer} + 1)!\, \left(N_{grid} - N_{mon} - N_{nmer} + n - 1\right)!}\,,$$

and thus, for $N_{mon} \gg n$, $N_{nmer} \gg 1$, $N_{grid} \gg N_{mon}, N_{nmer}$, the (per mole) change in configurational entropy is given by

$$\Delta S_{\substack{cf\ mon \\ to\ nmer}} = R \log\left(\frac{\Omega_{final}}{\Omega_{initial}}\right) = R \log\left[\frac{\left(\frac{N_{mon}}{N_{grid}}\right)^n}{\frac{N_{nmer}}{N_{grid}}}\right]. \qquad \text{Eq. S11}$$

From Eqs. S10 and S11 and the definition of the standard Gibbs free energy change ΔG^0 we have

$$R\, T \log\left[\frac{\left(\frac{N_{mon}}{N_{grid}}\right)^n}{\frac{N_{nmer}}{N_{grid}}}\right] = \Delta H - T\, \Delta S_{th} \equiv \Delta G^0{}_{\substack{mon \\ to\ nmer}}$$

at equilibrium, which can be rearranged to obtain

$$\frac{N_{nmer}}{N_{grid}} = \left(\frac{N_{mon}}{N_{grid}}\right)^n \exp\left(\frac{-\Delta G^0{}_{\substack{mon \\ to\ nmer}}}{R\, T}\right) \rightarrow c_{nmer} = A_0^{-1} (A_0\, c_{mon})^n \exp\left(\frac{-\Delta G^0{}_{\substack{mon \\ to\ nmer}}}{R\, T}\right), \qquad \text{Eq. S12}$$

as in Eq. 2 in the main text. The unit grid area A_0 (μm^2) permits expression of the experimental membrane protein concentration, $c_x = \frac{N_x}{A_0\, N_{grid}}$, as μm^{-2}.

Next, we specify $\Delta G^0{}_{\substack{mon \\ to\ nmer}}$ in Eq. S12 as a function of n through a simplified model of *nmer* formation. We consider first the special case of a very large cluster (formally, an infinitely large cluster), which we call

‘bulk phase’, at equilibrium with monomers on the grid. To express the equilibrium relationship between the monomer concentration and the bulk phase, we proceed as above, but for the transfer of one monomer from the grid to the bulk phase. The corresponding numbers of microstates on the grid are given by

$$\Omega_{initial} = \frac{N_{grid}!}{N_{mon}!\,(N_{grid} - N_{mon})!},$$

$$\Omega_{final} = \frac{N_{grid}!}{(N_{mon} - 1)!\,(N_{grid} - N_{mon} + 1)!}.$$

Furthermore, we assume that the bulk phase is a uniform (and infinite) collection of indistinguishable proteins for which addition (or subtraction) of a protein does not change its configurational entropy. We then have for the (per mole) change in configurational entropy of the system (grid plus bulk phase),

$$\Delta S_{\substack{cf\ mon \\ to\ bulk}} = R \log\left(\frac{\Omega_{final}}{\Omega_{initial}}\right) = R \log\left(\frac{N_{mon}}{N_{grid}}\right), \qquad N_{grid} \gg N_{mon}$$

and thus, for the monomer concentration at equilibrium with the bulk phase ($\Delta S_{tot} = 0$),

$$c_{\substack{mon \\ crit}} = A_0^{-1} \exp\left(\frac{\Delta G^0_{\substack{mon\ to \\ bulk}}}{R\,T}\right), \qquad \text{Eq. S13}$$

as in Eq. 3 in the main text, where we have again applied the relation $c_x = \frac{N_x}{A_0\,N_{grid}}$.

As described in the main text, the thermodynamic cycle in Figure 6 in the main text relates the direct transfer of *n* monomers from solution to the bulk phase (top) to a two-step process (bottom) in which a compact *nmer* is first formed, and then transferred to the bulk phase. Here, the incomplete bonding at the *nmer* boundary is proportional to the number of proteins at the perimeter of the *nmer* and thus, for simplicity, we set it proportional to $n^{0.5}$ with a proportionality constant equal to 1.0. At equilibrium, the two paths for transferring *n* proteins from the grid to the bulk phase must be energetically equivalent, which yields

$$\Delta G^0_{\substack{mon\ to \\ nmer}} = (n - n^{0.5})\,\Delta G^0_{\substack{mon\ to \\ bulk}}. \qquad \text{Eq. S14}$$

Substituting Eq. S14 into Eq. S12 we obtain for the distribution of *nmers*,

$$c_{nmer} = A_0^{-1}\,(A_0\,c_{mon})^n \exp\left[\frac{(n^{0.5} - n)\,\Delta G^0_{\substack{mon\ to \\ bulk}}}{R\,T}\right], \qquad \text{Eq. S15}$$

as in Eq. 5 in the main text. The term $n^{0.5}\Delta G^0_{\substack{mon\ to \\ bulk}}$ in Eqs. S14 and S15 is a boundary energy term subtracted from the standard free energy for transferring *n* monomers to the bulk phase ($n\,\Delta G^0_{\substack{mon\ to \\ bulk}}$). The *nmer* boundary energy is considered further in Appendix 3.

Appendix 3: The *nmer* boundary energy

Within the framework of statistical mechanics, the equilibrium *nmer* distribution in Eq. 2 in the main text corresponds to

$$N_n = \left(\frac{N_1}{z_1}\right)^n z_n \,, \qquad \text{Eq. S16}$$

where N_n denotes the total number of *nmers* in the membrane and z_n is the single-*nmer* partition function. Since our interest here lies in the self-assembly of HOTS from monomers, we use the monomeric state $n = 1$ as our reference state, for which Eq. S16 reduces to the identity $N_1 = N_1$. In Eq. S16 (and in Eq. 2 in the main text) we assume, for simplicity, that all interactions between *nmers* can be neglected, which amounts to the assumption that the membrane is dilute in the particular proteins forming HOTS. This is the scenario relevant for the experiments on HOTS described in the main text. We refer here to the proteins forming HOTS as 'protein units.' As in the main text, Eq. S16 assumes that HOTS with a given *n* cannot be distinguished and that $N_n \gg 1$ for all n so that $\ln N_n! \approx N_n \ln N_n - N_n$. Equation S16 can be obtained from the Helmholtz free energy of the system, by setting its partial derivatives with respect to N_n equal to zero under the constraint that the total number of protein units in the membrane takes a fixed value, which effectively fixes N_1/z_1 in Eq. S16 in the form of a Lagrange multiplier. For each *nmer*, the single-*nmer* partition function z_n in Eq. S16 includes contributions due to the *nmer*'s (binding) energy and translational degrees of freedom in the membrane. In principle, z_n may also include additional contributions due to, for instance, fluctuations in the HOTS shape.

To connect Eq. S16 to experiments on HOTS we need to specify the form of z_n. Considering the complexity of HOTS and of the cell membranes in which HOTS reside, z_n may involve many different (complicated) contributions with, in general, many unknown parameters. Our aim here is to make a minimal set of assumptions about the detailed molecular properties of HOTS, in order to extract the key (coarse-grained) features of HOTS and cell membranes underlying the measured HOTS distributions. Clearly, more complicated models of HOTS could be developed, which could be used to provide more detailed molecular descriptions of HOTS.

In our minimal model of HOTS self-assembly we assume that, effectively, the energy of an *nmer* only depends on the value of n and not, for instance, on the position of the HOTS within the membrane. Writing the *nmer* energy in the form $\epsilon(n)$, which means that the protein unit energy in the *nmer* is given by $\epsilon(n)/n$, we then have

$$z_n = \Omega\, e^{-\beta\, \epsilon(n)} \,, \qquad \text{Eq. S17}$$

where Ω denotes the number of distinct configurational microstates accessible to the HOTS and $\beta = 1/k_B T$, in which k_B denotes Boltzmann's constant and T denotes the system temperature. For simplicity, we assume in the main text that Ω takes the same value for all n, $\Omega = A/A_0$ with a constant A_0. A more detailed model of HOTS would allow for a dependence of A_0 on n. Note that each $\epsilon(n)$ is only defined up to an additive constant, which must be chosen so as to obtain the correct energy changes in all *nmer* assembly/disassembly reactions. Using, again, $n = 1$ as our reference state we set here $\epsilon(1) = 0$, which allows us to interpret $\epsilon(n)$ as the energy of forming an *nmer* from n monomers. Equations S16 and S17 reduce to Eq. 2 in the main text upon identifying $\epsilon(n) \equiv \Delta G^0_{\substack{mon\,to \\ nmer}}/N_A$, where N_A denotes the Avogadro constant.

At the most basic level, the *nmer* energy $\epsilon(n)$ involves a bulk contribution arising from favorable interactions between protein units in the interior of HOTS. This negative bulk energy is expected to be proportional to the number of protein units in *nmers*, and to dominate the *nmer* energy in the large-n limit. Furthermore, the protein units forming the *nmer* perimeter can only interact favorably with other protein units in the *nmer* interior or along the *nmer* perimeter, which is expected to be energetically unfavorable and to yield a positive contribution to $\epsilon(n)$ proportional to the HOTS perimeter (see also Figure 6 in the main text). A simple model of $\epsilon(n)$ is thus provided by

$$\epsilon(n) = \epsilon_b(n-1) - \epsilon_p(n^\delta - 1), \qquad \text{Eq. S18}$$

where the cohesive interaction strength $\epsilon_b < 0$ in the bulk of HOTS, $\epsilon_p < 0$ parameterizes the energy penalty associated with the HOTS perimeter, and we expect $0 < \delta < 1$. In the main text we assume, for simplicity, that the HOTS perimeter is proportional to the square-root of the HOTS area, i.e., n, resulting in $\delta = 1/2$ in Eq. S18. Values of δ other than $1/2$, different functional forms of the boundary energy, or more complicated expressions for $\epsilon(n)$ could arise, for instance, from HOTS shapes that may, for example, be fractal or vary with n, directional interactions between protein units, or a dependence of the strength of protein unit interactions in *nmers* on n. Instead of employing an analytic expression for $\epsilon(n)$ as in Eq. S18, one could use bond counting to model $\epsilon(n)$. Interactions of protein units with other membrane components may also affect $\epsilon(n)$. We regard Eq. S18 with $\epsilon_b = \epsilon_p$ and $\delta = 1/2$ as the most straightforward model of the HOTS energy, and therefore focus, in the main text, on this functional form of $\epsilon(n)$. Below, we consider a few generalizations of the HOTS boundary energy considered in the main text.

Allowing for $\epsilon_b \neq \epsilon_p$ in Eq. S18

Since the ϵ_p-term in Eq. S18 arises from a reduction in favorable protein unit interactions at HOTS boundaries we expect ϵ_b and ϵ_p to take comparable values. In the main text we therefore use the functional form of $\epsilon(n)$ in Eq. S18 with, for simplicity, $\epsilon_b = \epsilon_p = \Delta G^0_{\substack{mon\ to \\ bulk}}/N_A$. The detailed relation between ϵ_b and ϵ_p depends on, for instance, the HOTS shape and on the protein unit interactions in HOTS. For illustration, consider the idealized case of perfectly circular HOTS shapes. In this case we would have $\epsilon_p = b\,\epsilon_b$ with $b = \sqrt{\pi}$ if the number of protein units along the *nmer* perimeter is equal to the perimeter length divided by the square-root of the protein unit area, and if the protein unit energy along the *nmer* perimeter is one-half the protein unit energy in the *nmer* interior.

Note that the values of $\Delta G^0_{\substack{mon\ to \\ bulk}}$ extracted in the main text determine ϵ_p through $\epsilon_p = \Delta G^0_{\substack{mon\ to \\ bulk}}/N_A$, while the ϵ_b-term in Eq. S18 can effectively be absorbed into the Lagrange multiplier ν fixing the total number of protein units in the membrane, c_{tot}. In particular, in terms of Eqs. S16–S18, Eq. 5 in the main text corresponds to

$$c_{nmer} = c_{mon}\,\nu^{n-1}\,e^{\beta\,\epsilon_p\,(\sqrt{n}-1)}, \qquad \text{Eq. S19}$$

where we set $\nu = c_{mon}\,A_0\,e^{-\beta\,\epsilon_b}$ and $\delta = 1/2$. Because c_{mon} is directly measured in experiments and ν is determined by the measured total concentration of protein units in the membrane, ϵ_p is the only unknown parameter in Eq. S19. The value of ϵ_p is expected to depend on the particular protein species under consideration but, at least to a first approximation, not on the expression level of that protein. We therefore use the same value of ϵ_p for all curves in Figure 8G in the main text. Since Eq. 5 in the main text has the same form as Eq. S19, the comparisons between measured and predicted HOTS distributions in the main text are not affected if $\epsilon_b \neq \epsilon_p$. However, because $A_0 = \frac{\nu}{c_{mon}} e^{\beta\,\epsilon_b}$, estimates of A_0 and, hence, of the configurational length scale obtained from such comparisons do depend on how ϵ_b is related to ϵ_p. In particular, if $|\epsilon_b| < |\epsilon_p|$, as in the idealized case of perfectly circular *nmer* shapes considered above, the values of A_0 obtained in the main text would underestimate A_0.

Allowing for $\delta \neq 1/2$ in Eq. S18

Using Eq. S18 with $0 < \delta < 1$, we can proceed as in the main text to fit the predicted HOTS distributions to experimental measurements of c_{nmer}. We thus find similarly good agreement between predicted and measured HOTS distributions as obtained in Figure 8G in the main text with $\delta = 1/2$. But, depending on the value of δ considered, somewhat different fitted values of ϵ_p and estimates of A_0 are obtained. To

illustrate this point we show, below, fitted values of ϵ_p, as well as the corresponding estimates of the average A_0 obtained with $\epsilon_b = \epsilon_p$, $\bar{A}_0$, as a function of δ in Eq. S18:

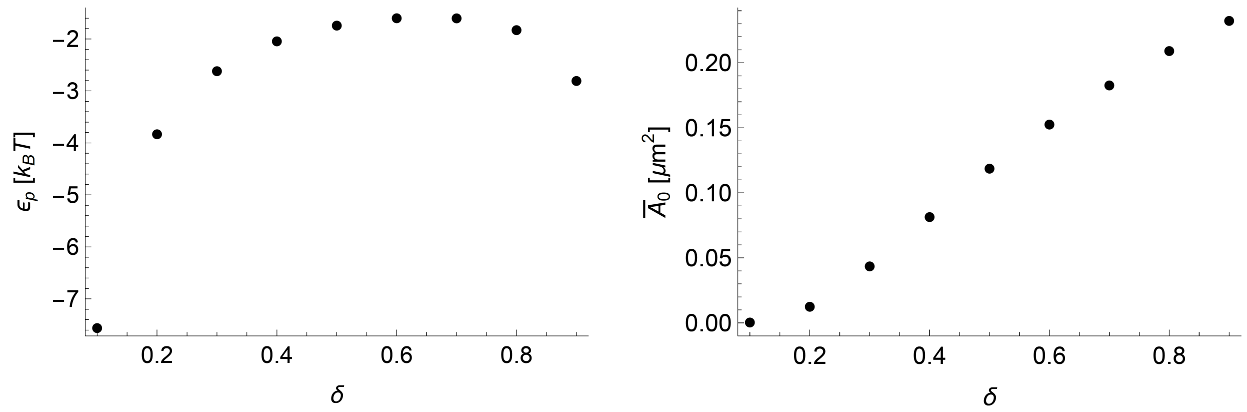

At each δ, $\bar{A}_0$ corresponds here to the average A_0 across the six data sets in Figure 8G in the main text. As in the main text, we take ϵ_p to be independent of the protein expression level and therefore have, at each δ, a single value of ϵ_p for all six data sets in Figure 8G in the main text. The above results suggest that ϵ_p changes non-monotonically with δ, while $\bar{A}_0$ increases with δ. At small δ, (relatively) small values of $\bar{A}_0$ are obtained. For instance, for $\delta = 0.1$ we have $\bar{A}_0 \approx 300\ \mathrm{nm}^2$, while for larger δ we have, roughly, $\bar{A}_0 \sim 0.1\ \mu\mathrm{m}^2$.

Allowing for a logarithmic boundary energy term in Eq. S18

Beyond the functional form of $\epsilon(n)$ considered in Eq. S18 one may, for instance, allow for a boundary energy term that grows logarithmically with n,

$$\epsilon(n) = \epsilon_b(n-1) - \theta \log(n)\,, \qquad \text{Eq. S20}$$

where $\theta < 0$, which results in the *nmer* distribution

$$c_{nmer} = c_{mon}\ \nu^{n-1}\ n^{\beta\,\theta} \qquad \text{Eq. S21}$$

with, again, $\nu = c_{mon}\ A_0\ e^{-\beta\,\epsilon_b}$. Fitting Eq. S21 to the data in Figure 8G in the main text yields $\theta \approx -0.75\ k_BT$, suggesting a power-law exponent equal to (minus) $3/4$ in Eq. S21. With $\theta \approx -0.75\ k_BT$, Eq. S20 produces, within experimental uncertainty, a similarly good fit to the data in Figure 8G in the main text as Eq. S18 with $0 < \delta < 1$. Assuming, again, that ϵ_b and the boundary energy θ take comparable values, the data in Figure 8G in the main text yield $0.16\ \mu\mathrm{m}^2 \lesssim A_0 \lesssim 0.51\ \mu\mathrm{m}^2$ in Eq. S21. Note, however, that Eq. S21 with $\theta \approx -0.75\ k_BT$ does not, at $\nu = 1$, produce a convergent sum over $n\ c_{nmer}$ from $n = 1$ to $n \to \infty$, in contrast to Eq. S19 (see also Eqs. 6 and 7 in the main text). Thus, with $\theta \approx -0.75\ k_BT$ the model of the *nmer* energy in Eq. S20 does not predict a transition to a bulk phase, in apparent contradiction to the experiments described in the main text. If θ is restricted to $\theta < -2\ k_BT$ in Eq. S20 so as to yield a convergent (positive) sum over $n\ c_{nmer}$ at $\nu = 1$, Eq. S21 only seems to allow for a poor fit to the data in Figure 8G in the main text.

In summary, the above calculations illustrate how, through reversible self-assembly, many different models of HOTS can give rise to similar HOTS distributions. Different mechanisms for HOTS self-assembly can yield different predictions regarding detailed, but potentially important, features of the HOTS distribution, such as the form of the limiting distribution as saturation is approached, and whether a bulk phase can form. The above calculations also illustrate how the value of A_0 implied by the measured HOTS distributions depends on the particular model of HOTS used, making A_0 a model-dependent parameter.

Appendix 4: Dependence of the *nmer* distribution on A_0 and $\Delta G^0_{\substack{mon\ to\\bulk}}$

This appendix illustrates how the predicted HOTS distribution c_{nmer} in Eq. 5 in the main text with the constraint in Eq. 8 in the main text depends on the values of the parameters A_0 and $\Delta G^0_{\substack{mon\ to\\bulk}}$. In the figure shown below, we replot the data and fitted curve from Figure 8G in the main text for a total protein concentration $c_{tot} \approx 3.76\ \mu\mathrm{m}^{-2}$ (black data points and black curves):

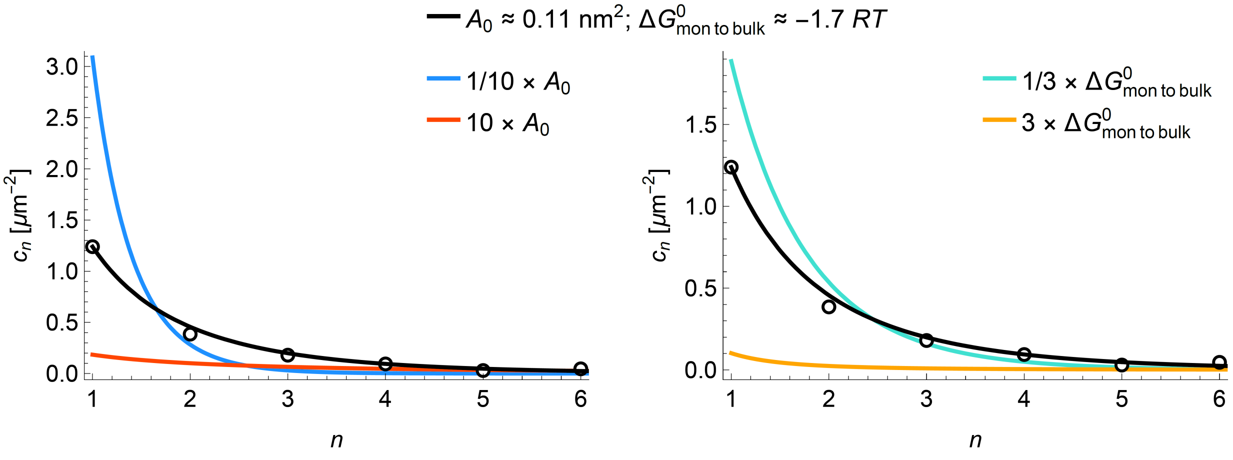

In the left panel of the above figure we decreased and increased A_0 by factors of $1/10$ and 10 compared to the value of A_0 used for the black curves, respectively, while keeping the value of $\Delta G^0_{\substack{mon\ to\\bulk}}$ fixed to the value used for the black curves, and let c_{mon} vary so as to satisfy the constraint in Eq. 8 in the main text. Conversely, in the right panel we decreased and increased the magnitude of $\Delta G^0_{\substack{mon\ to\\bulk}}$ by factors of $1/3$ and 3 compared to the value of $\Delta G^0_{\substack{mon\ to\\bulk}}$ used for the black curves, respectively, while keeping the value of A_0 fixed to the value used for the black curves, and let c_{mon} vary so as to satisfy the constraint in Eq. 8 in the main text. We find that, for a fixed c_{tot}, the shape of the HOTS distribution changes qualitatively with the values of A_0 and $\Delta G^0_{\substack{mon\ to\\bulk}}$. In particular, larger A_0 and, hence, a larger configurational length scale $\sqrt{A_0}$, as well as larger magnitudes of $\Delta G^0_{\substack{mon\ to\\bulk}}$ produce broader HOTS distributions. A large enough $\sqrt{A_0}$ therefore allows, at a given (small) c_{tot}, for broad HOTS distributions even at small magnitudes of $\Delta G^0_{\substack{mon\ to\\bulk}}$, as found for the data in Figs. 8 and 9 in the main text.

Appendix 5: The under-labeling problem

For protein counting in this study, through screening we selected primary antibodies that are specific to the protein target and have a low background signal. But a question that undoubtedly comes to the reader's mind is, to what extent will under-labeling affect our ability to detect the features that characterize oligomerization? To answer this question, we consider the following hypothetical case in which the membrane proteins are partially labelled in a random manner with probability P_{label}. We seek the observed cluster size distribution of labels, $f_{label}(n)$, given an underlying membrane protein cluster size distribution $c_{nmer}(n)$, expressed in Eq. 5 in the main text as

$$c_{nmer}(n) = \frac{1}{A_0}(A_0\, c_{mon})^n \exp\left[\frac{(n^{0.5} - n)\Delta G^0_{\substack{mon\ to\\ bulk}}}{R\,T}\right]. \qquad \text{Eq. S22}$$

To obtain the distribution of labels, note that the 'monomer' bin will contain labelled monomers plus protein dimers with one label plus protein trimers with one label, etc. The 'dimer' bin will contain protein dimers with two labels plus protein trimers with two labels plus protein tetramers with two labels, etc. One immediately deduces that the cluster size distribution of labels is given by

$$f_{label}(n) = \sum_{x=n}^{\infty} \binom{x}{n} P_{label}^n\,(1 - P_{label})^{x-n} c_{nmer}(x)\ . \qquad \text{Eq. S23}$$

When we analyze our data in this study, we count the *nmers*, divide by the membrane area, and fit the experimental distribution to Eq. S22 under the constraint expressed in Eq. 8 in the main text,

$$c_{tot} = \sum_{n=1}^{n_{max}} n\, c_{nmer}(n)\,. \qquad \text{Eq. S24}$$

Because c_{mon} and c_{tot} are measured, Eq. S22 combined with the constraint in Eq. S24 allow us to fit $\Delta G^0_{\substack{mon\ to\\ bulk}}$, from which A_0 is determined.

As an exercise here, we use Eq. S22 with 'true' values of $\Delta G^0_{\substack{mon\ to\\ bulk}}$, A_0, and c_{mon} to generate 'data' for a protein cluster size distribution (symbols):

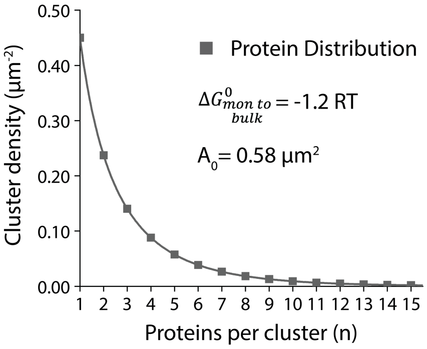

We note that the true values for this protein distribution are near those determined for M2Rs in HL-1 cells. When analyzing these data using Eqs. S22 and S24 as described, we of course obtain the true values of $\Delta G^0_{\substack{mon\ to\\ bulk}}$ and A_0 (curve). Next, we use Eq. S23 with $P_{label} = 0.5$ to generate a corresponding size distribution of labels (symbols):

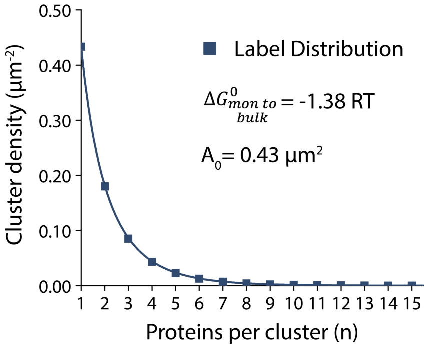

Now we pretend that the labels are the proteins, i.e., we assume that the labeling is complete, and again fit Eq. S22 with Eq. S24 to the distribution of labels (curve) to obtain estimates for $\Delta G^0_{\substack{mon\ to \\ bulk}}$ and A_0. We see that even with severe (50%) under-labeling, the estimates for $\Delta G^0_{\substack{mon\ to \\ bulk}}$ and A_0 deviate from the true values, but they are not very far off. This supports the position that potential under-labeling does not prevent us from capturing the general features of HOTS distributions.

Appendix 6:

HOTS and bulk phase cluster formation through oligomerization in reconstituted membranes

If clusters form through self-oligomerization, then they should occur in the absence of other cellular components. To see if this is so, we studied GIRK and another K^+ channel, Kv2.1, because they are larger than M2R and thus easier to visualize and count. When reconstituted into synthetic lipid membranes, both channels form compact clusters (Figure 10). Negative stain EM images contain single membrane sheets with channels (Figure 10A, C). Cryo-EM images contain large, flattened vesicles (Figure 10E, F). Two membranes are present in the latter, but for Kv2.1 the top and bottom membranes are distinguishable owing to the recognizable handedness of the channels and the known orientation with which they insert into vesicles. A caveat on the cluster size distributions in reconstitution experiments is that we do not have very large single membrane sheets like in unroofed cell membranes. Since different vesicles may have different protein concentrations, we consider these distributions qualitative. The result, however, is clear: both K^+ channels form cluster size distributions showing the key properties of HOTS size distributions, as well as much larger, presumably bulk phase clusters (Figure 10B, D, G).

In many cases, the arrangement of membrane proteins in HOTS is highly irregular. An example of this is shown for Kv2.1, where four pairs of adjacent neighbors inside clusters exhibit non-uniform relative protein orientations (Figure 10H). Such irregularity is explicable if these proteins can oligomerize either through multiple distinct contacts, or if the contacting sites are formed through disordered segments that are flexible. Importantly, neighboring proteins inside HOTS are close enough to make direct contacts through structural elements or extended loops (Figure 4E).

Single particle tracking in reconstituted membranes offers another way to assess the ability of a protein to self-oligomerize. To this end, M2R was labeled with a fluorescent nanobody directed against a C-terminal ALFA tag and recorded in the freestanding bilayer microscope in synthetic membranes without other proteins (Figure 10I) (1, 2). The dwell-time histogram graphs the number of PSF overlap events against the overlap duration (Figure 10J). The histogram is biexponential, with a short duration, ~ 58 ms, corresponding to the time we expect PSFs from two non-interacting proteins to overlap coincidentally. The longer time constant in the biexponential histogram, ~ 300 ms, corresponds to an event that keeps two M2Rs together for a period longer than the no-interaction coincidence time. We interpret this event to be a successful encounter in which two M2Rs interact with each other. In this experiment, the concentration of M2Rs is very low ($\sim 0.1 - 0.2\ \mu\text{m}^{-2}$, with high labeling efficiency) so that the HOTS size distribution will be dominated by monomers, with rare dimers and even rarer higher-order oligomers. The detection of pairwise interactions between two M2Rs in a reconstituted system demonstrates their oligomerization and provides a measure of transience. These results inspired our next experiments on the dynamic properties of HOTS in cells.

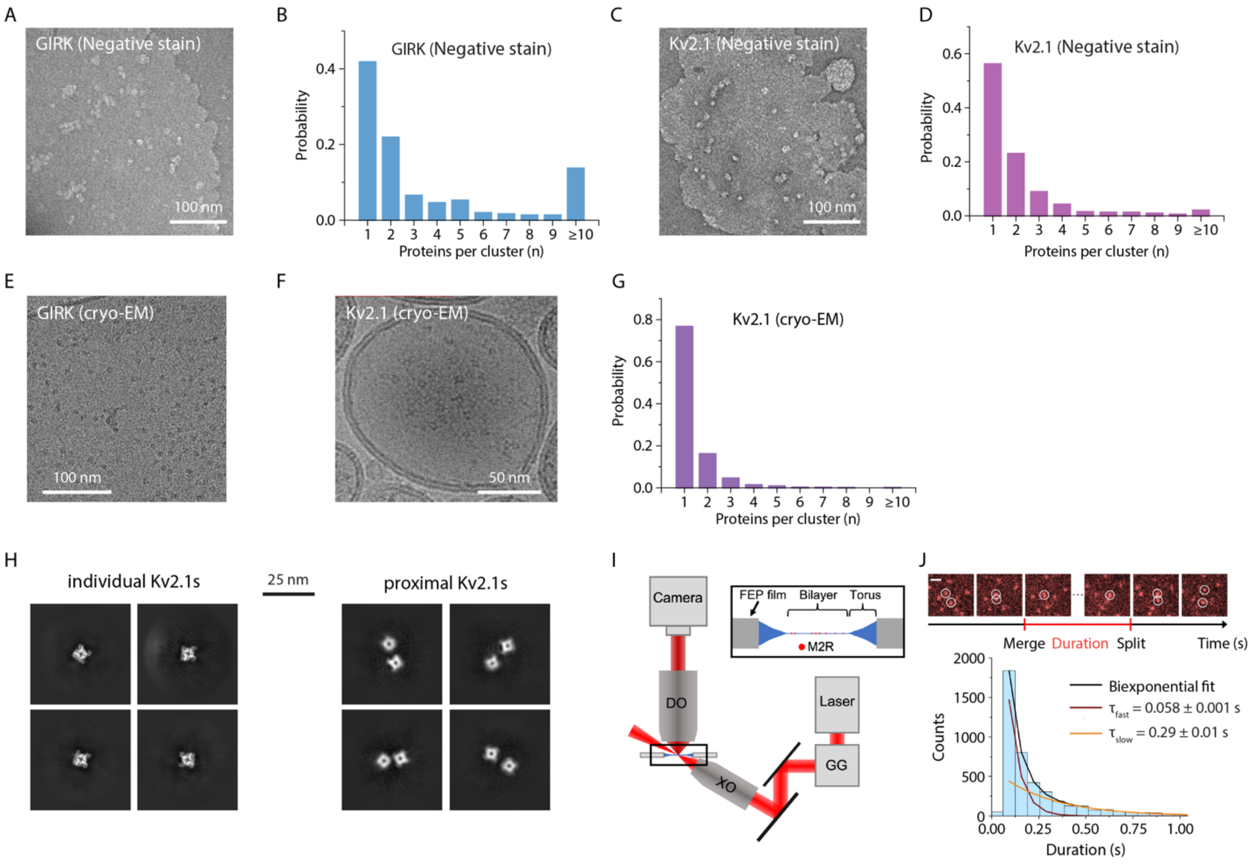

Figure 10. Protein interactions in reconstituted membranes.

(A) Representative image of a flattened POPC membrane containing reconstituted GIRK channels (white particles) visualized by negative stain electron microscopy. The edge of the membrane sheet is visible on the right-hand side of the image. Proteoliposomes were burst on a carbon-coated grid and stained with uranyl acetate. Scale bar: 100 nm.

(B) Cluster-size distribution of GIRK channels in these membranes constructed from 1017 individual channels. Here as in (D) and (G) the histogram is normalized by the total channel number and thus graphed as probability.

(C) Negative stain image of reconstituted Kv2.1 channels and (D) the corresponding cluster-size distribution constructed from 1056 individual channels.

(E) Representative cryogenic electron microscopy (cryo-EM) image of GIRK channels reconstituted into POPC vesicles. Two membranes (the top and bottom membranes of the vesicle) span the entire view of the image and top/bottom views of channels are visible as dark particles. GIRK channels reconstitute in both directions (inside-in and inside-out) in the two membranes, which precludes cluster analysis as it is not possible to determine whether particles originate from the top or the bottom membrane.

(F) Cryo-EM image of Kv2.1 channels reconstituted into POPC vesicles. Kv2.1 reconstitutes exclusively in the inside-in orientation. This allows identification and separation of particles on the top and bottom membranes through analysis of handedness in 2-dimensional class averages of the particles, which in turn permits cluster size distribution analysis.

(G) Cluster-size distribution of Kv2.1 inferred from cryo-EM analysis. The quantitatively different cluster size distributions in panels (D) and (G) likely reflect the inhomogeneity of protein concentrations in different vesicles, rendering these distributions qualitative.

(H) Representative 2-dimensional class averages of individual (left) and proximal (right) Kv2.1 channels. Channels within clusters pack against each other at different angles, indicating that the protein-protein interactions can occur in different ways (or involve non-structured protein regions).

(I) Simplified schematic of the freestanding bilayer microscope (1). A 660 nm laser is fed to a galvo-galvo (GG) scanner conjugated to the back focal plane of the excitation objective (XO) that focuses the laser onto a freestanding bilayer that contains fluorescently labeled M2R. The emission of fluorophores is collected by a detection objective (DO) and recorded using a sCMOS camera. Insert: Schematic of a freestanding bilayer showing the stabilizing boundary of the bilayer (torus) and the supporting material (FEP).

(J) M2R interactions in freestanding bilayers. Top: Example of colocalization between two fluorescently labeled M2R in a freestanding bilayer made of POPE:POPG 3:1 (weight ratio) lipids. Bottom: Frequency histogram of the duration of M2R colocalizations and bi-exponential fit (black line). The fast (red) and slow (orange) components of the fit decay with time constants (τ) of 0.058 ± 0.001 s and 0.29 ± 0.01 s, respectively. The M2R concentration in the membrane was 0.1 – 0.2 μm^{-2} and labeling efficiency was high (~ 40%).

The dynamical nature of HOTS

Figure 11 compares on the same spatial scale an EM micrograph from an unroofed HL-1 cell with gold labeled M2R (Figure 11A) and trajectories from a 2-minute video recording of Cy3-B-telenzepine labeled M2R in a live HL-1 cell (Figure 11B). The trajectories show that M2R proteins diffuse over micrometer distances, periodically appear more immobilized, and then continue diffusing. The tracks with distinct colors show that different M2Rs become immobilized in similar regions of the membrane. In Figure 11C we compare nearest neighbor distance histograms for these positions of relative immobilization to the corresponding histograms obtained from clusters ($n \geq 3$) identified in EM montages from HL-1 cells. The parsimonious explanation for their similarity is that M2Rs tend to move more slowly or become immobilized once they enter a cluster. Consistent with this explanation, in PtK2 cells in which we analyze M2R diffusion at different levels of expression, the average diffusion coefficient decreases, and the immobilized fraction increases when the M2R density is increased (See SI Appendix, Fig. S7). The picture we have in mind is that clusters self-assemble out of diffusing protein units, but they are dynamic, because the interactions between the individual protein units are weak. Clusters appear, grow, melt away, and constantly exchange units, as we saw in the toy model simulation (see SI Appendix, Movie S5).

For large bulk phase protein clusters, protein unit exchange between clusters can be visualized directly. M2R containing an extracellular HA tag were over-expressed in PtK2 cells and labeled with a fluorescent antibody. This primary antibody was then sparsely labeled with a secondary antibody containing a single 40 nm gold particle, visible under differential interference contrast microscopy as a PSF-sized circular shadow (3, 4). A movie shows the diffusion of individual M2Rs superimposed on a fluorescent image of the same region of membrane (see SI Appendix, Movie S6). M2Rs are abundant in this membrane, apparent as a diffuse green background fluorescence and as large (micrometer-sized) intense green-fluorescent patches. The large, intense patches presumably represent bulk phase M2R clusters expected at this level of M2R expression. The movie proceeds at a rate of 150 ms per frame. Individual gold-labeled M2R in the diffuse green region move randomly, stopping and starting briefly and thus, presumably, interact with HOTS (corresponding to the immobilized occurrences in Figure 11B), but when they contact large bulk phase clusters, they tend to become immobilized for longer periods of time. Some M2R embedded inside the bulk phase clusters remain immobilized for the length of the movie.

As in the reconstituted, freestanding membrane system described above (Figure 10I, J), we observe individual M2R oligomerization events in HL-1 cell membranes while recording trajectories like those shown in Figure 11B. The panels in Figure 11D document an association and subsequent dissociation event between two PSFs from Cy3-B-telenzepine labeled M2R in HL-1 cells, corresponding to natural expression levels. The dwell-time histogram from many such events is biexponential, like in the reconstituted system. In the cell membrane, however, the time constants for both components are longer. There are several reasons for this. First, the M2R diffusion coefficient for Brownian motion is about $1.0\ \frac{\mu\mathrm{m}^2}{s}$ in the freestanding, reconstituted membrane and about $0.05 - 0.10\ \frac{\mu\mathrm{m}^2}{s}$ in the cell membrane. The slower diffusion in the cell membrane means that overlaps between PSFs persist longer even when coincident M2Rs do not interact, thus lengthening the shorter time constant. Second, molecular crowding in the cell membrane not only affects diffusion rates but also alters the thermodynamics of oligomerization reactions, favoring the oligomerized state and likely lengthening the longer time constant (5-8). Finally, a PSF in the reconstituted system, where the M2R density is $0.1 - 0.2\ \mu\mathrm{m}^{-2}$, nearly always represents a single M2R and therefore encounters are bimolecular. By contrast, in the HL-1 plasma membrane the M2R density is more than 10 times higher and, as described above, other crowding proteins in the cell membrane compared to the freestanding bilayer likely favor oligomerization of M2R. While single PSFs are observable because we under-label the proteins and/or photobleach the fluorophores, at these higher protein concentrations PSFs often mark a cluster rather than a single M2R. Kinetically, successful encounter events involving at least one cluster (as will often be the case in the cell membrane) will not be the same as successful encounter events between individual M2Rs (as will be the case in the freestanding bilayer).

The essential point is, oligomerization of weakly interacting proteins is a dynamic process and HOTS, as their name indicates, are transient objects that rapidly exchange their protein components with each other.

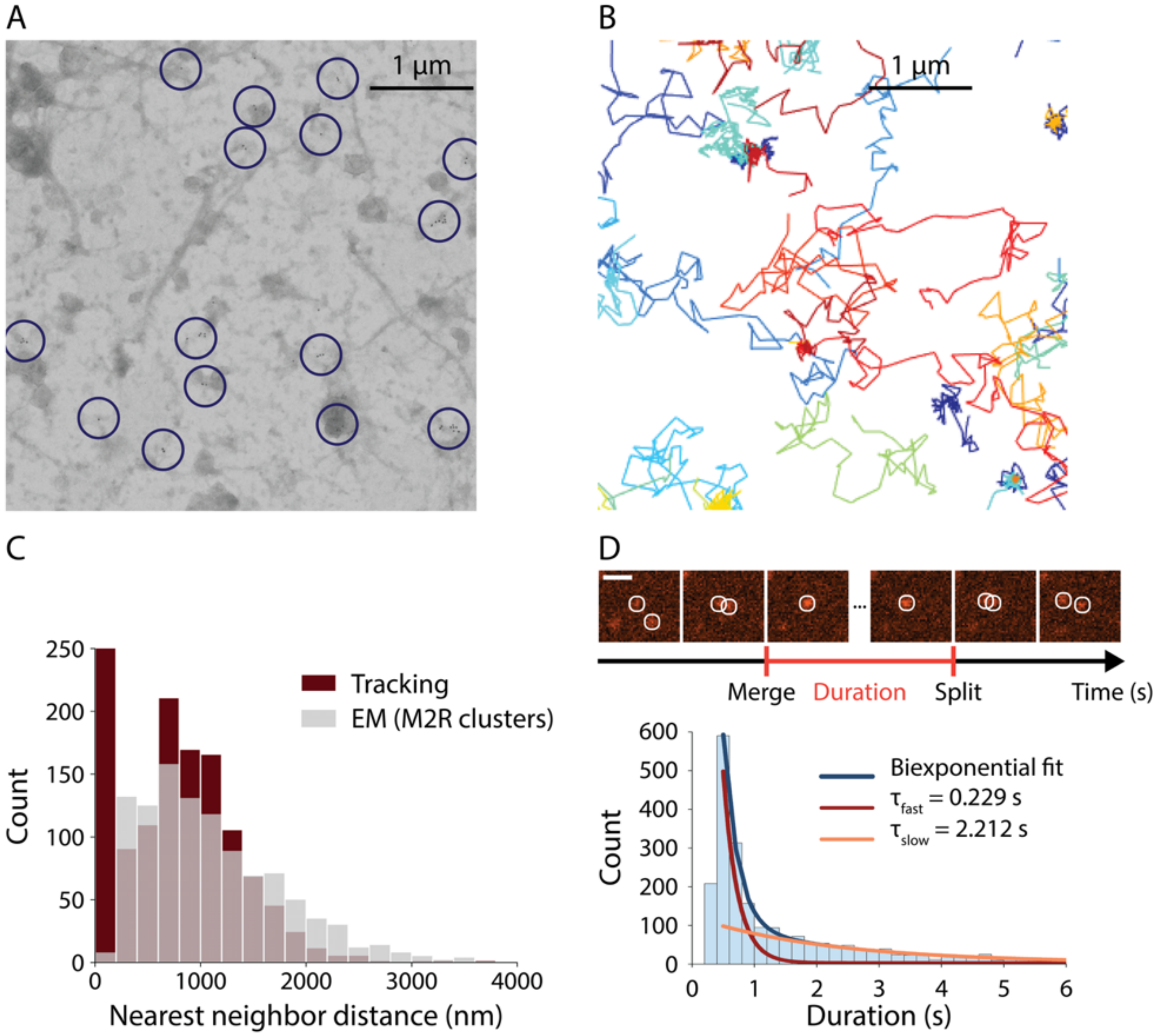

Figure 11: HOTS are dynamic.

(A) Representative negative stain electron micrograph of an unroofed HL-1 cell with M2R labeled with 18 nm gold particles. Locations of gold particles are highlighted by circles. Scale bar: 1 µm.

(B) Representative trajectories from a 2-minute video recording of Cy3B–telenzepine labeled M2R in HL-1 cells are shown. Different colors indicate different trajectories. Scale bar: 1 µm.

(C) Nearest neighbor distance histograms of M2R cluster (n ≥ 3) centroids from the electron micrographs (grey) and from the mean coordinates of each immobile trajectory (brown) in HL-1 cells are shown.

(D) An example of two Cy3B–telenzepine labeled M2Rs (or M2R clusters) colocalizing in HL-1 cells is shown (top) and the colocalization time distribution (bottom) is fit with a biexponential function (blue curve). The fast component (red curve) presumably corresponds to chance (noninteracting) colocalizations and the slow component (orange curve) to successful encounters (oligomerization events) between M2Rs in HL-1 cells. Scale bar: 1 µm.

References for Appendix 6

1. G. Perez-Mitta, Y. Sezgin, W. Wang, R. MacKinnon, Freestanding bilayer microscope for single-molecule imaging of membrane proteins. Sci Adv 10, eado4722 (2024).
2. H. Gotzke et al., The ALFA-tag is a highly versatile tool for nanobody-based bioscience applications. Nat Commun 10, 4403 (2019).
3. Y. Wu, M. R. K. Ali, K. Chen, N. Fang, M. A. El-Sayed, Gold nanoparticles in biological optical imaging. Nano Today 24, 120-140 (2019).
4. A. Kusumi, Y. Sako, M. Yamamoto, Confined lateral diffusion of membrane receptors as studied by single particle tracking (nanovid microscopy). Effects of calcium-induced differentiation in cultured epithelial cells. Biophysical Journal 65, 2021-2040 (1993).
5. A. P. Minton, The effect of volume occupancy upon the thermodynamic activity of proteins: some biochemical consequences. Mol Cell Biochem 55, 119-140 (1983).
6. R. J. Ellis, Macromolecular crowding: obvious but underappreciated. Trends Biochem Sci 26, 597-604 (2001).
7. C. Alfano et al., Molecular Crowding: The History and Development of a Scientific Paradigm. Chem Rev 124, 3186-3219 (2024).
8. C. A. Haselwandter et al., Role of the configurational length scale in HOTS formation. In preparation.

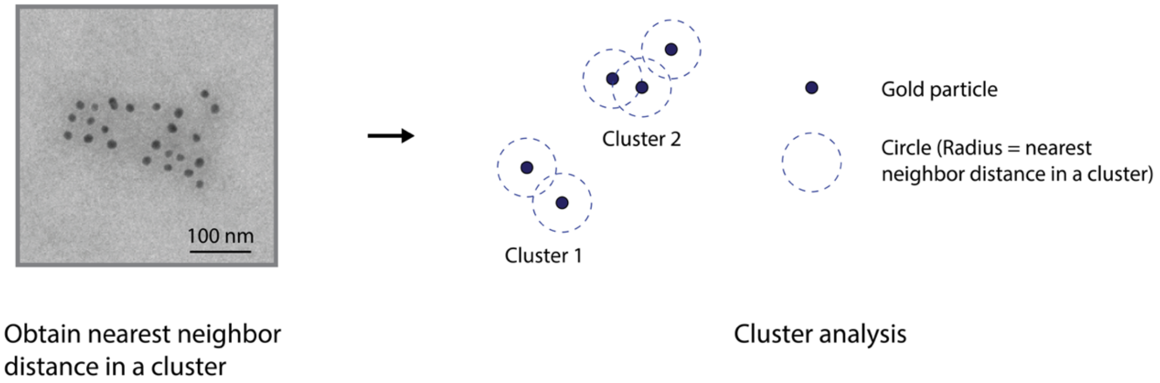

Fig. S1. Definition of a cluster in cell membranes. Nearest neighbor distance analysis was first performed to obtain the nearest neighbor distance of gold particles in a cluster. A circle of radius equal to this nearest neighbor distance was then drawn around each gold particle. Gold particles are defined to be in one cluster if their circles overlap.

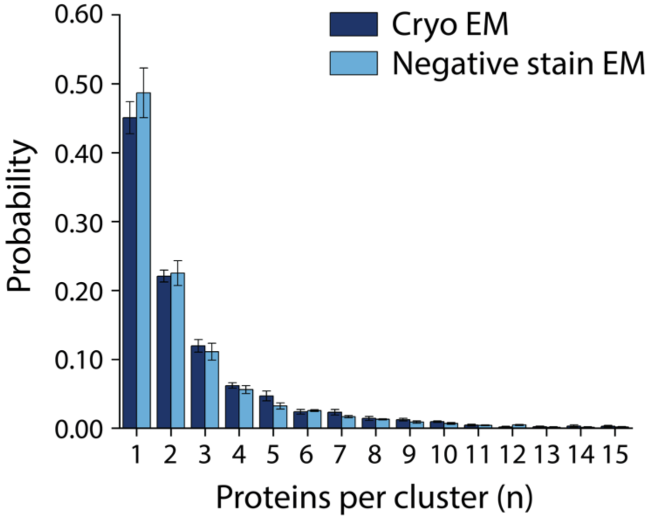

Fig. S2. Analysis of the M2R cluster size distribution by negative stain and cryo-EM using 18 nm gold labels. Normalized M2R (labeled with 18 nm gold particles) cluster size distributions in HL-1 cells from montages imaged by negative stain electron microscopy (light blue) and cryo-electron microscopy (dark blue). Non-specific gold particle labeling was estimated from CHO cells without heterologous expression and subtracted before normalization. Both samples were prepared at the same time. Data represent means and standard errors from n electron microscope montages. n = 6 for cryo-electron microscopy (6,871 gold particles being analyzed) and n = 5 for negative stain electron microscopy (10,080 gold particles being analyzed).

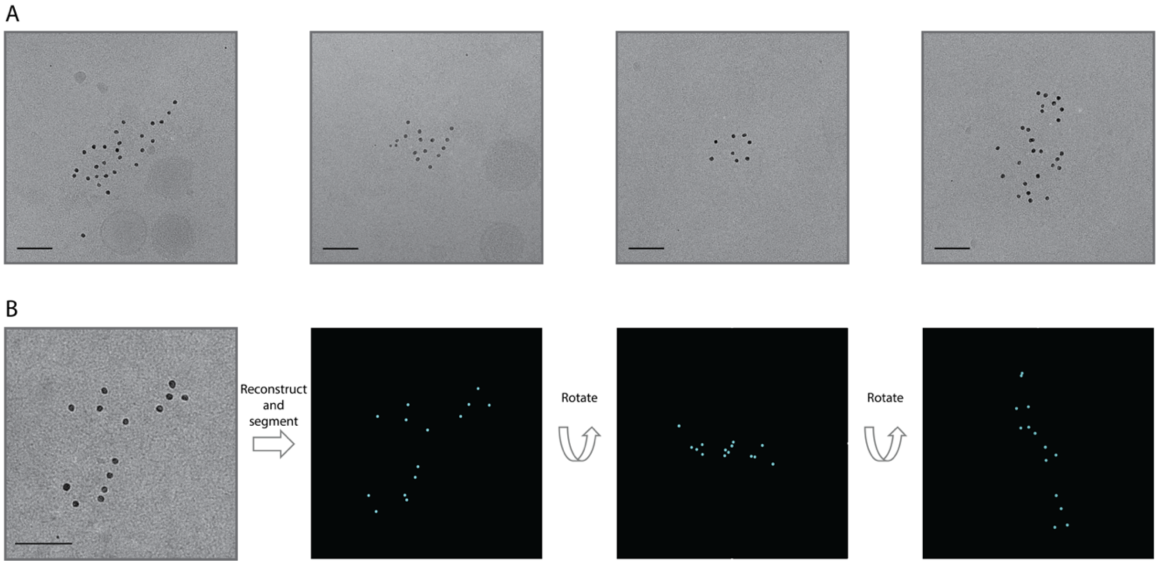

Fig. S3. Tomographic analysis of the shape of M2R clusters in HL-1 cell membranes. (A) Representative 0-degree tilt cryo-electron micrographs of M2R clusters. Proteins are labeled with 12 nm gold particles. Scale bars: 100 nm. (B) Gold particles in most M2R clusters are roughly in the same plane. A representative 0-degree tilt micrograph of an M2R cluster (left), its top view and two orthogonal views of segmented gold particles (cyan) in the reconstruction. Scale bars: 100 nm.

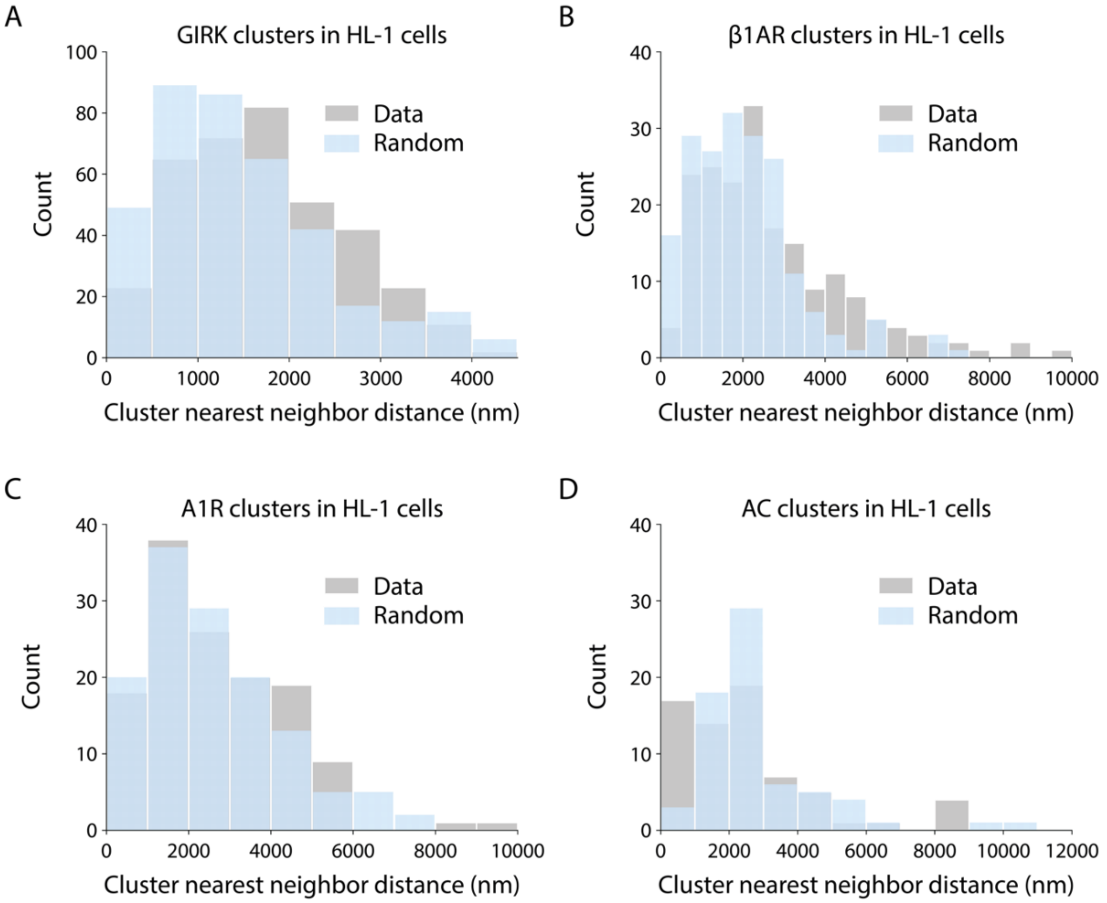

Fig. S4. Clusters of four proteins are randomly distributed on the surface of HL-1 cells. (A) Nearest neighbor distance histograms of GIRK cluster centroids (grey) in HL-1 cells and randomly distributed particles (blue). Clusters are defined to have at least three gold particles. Cluster centroids are calculated by averaging the x and y coordinates of all gold particles in each cluster. In the random simulation, the number of generated particles matches the number of cluster centroids, and particles only distribute within unroofed membranes. Proteins are labeled with 18 nm gold particles. (B) Nearest neighbor distance histograms of β1AR cluster centroids (grey) in HL-1 cells and randomly distributed particles (blue) as in panel (A). Proteins are labeled with 18 nm gold particles. (C) Nearest neighbor distance histograms of A1R cluster centroids (grey) in HL-1 cells and randomly distributed particles (blue) as in panel (A). Proteins are labeled with 18 nm gold particles. (D) Nearest neighbor distance histograms of AC cluster centroids (grey) in HL-1 cells and randomly distributed particles (blue) as in panel (A). Proteins are labeled with 18 nm gold particles.

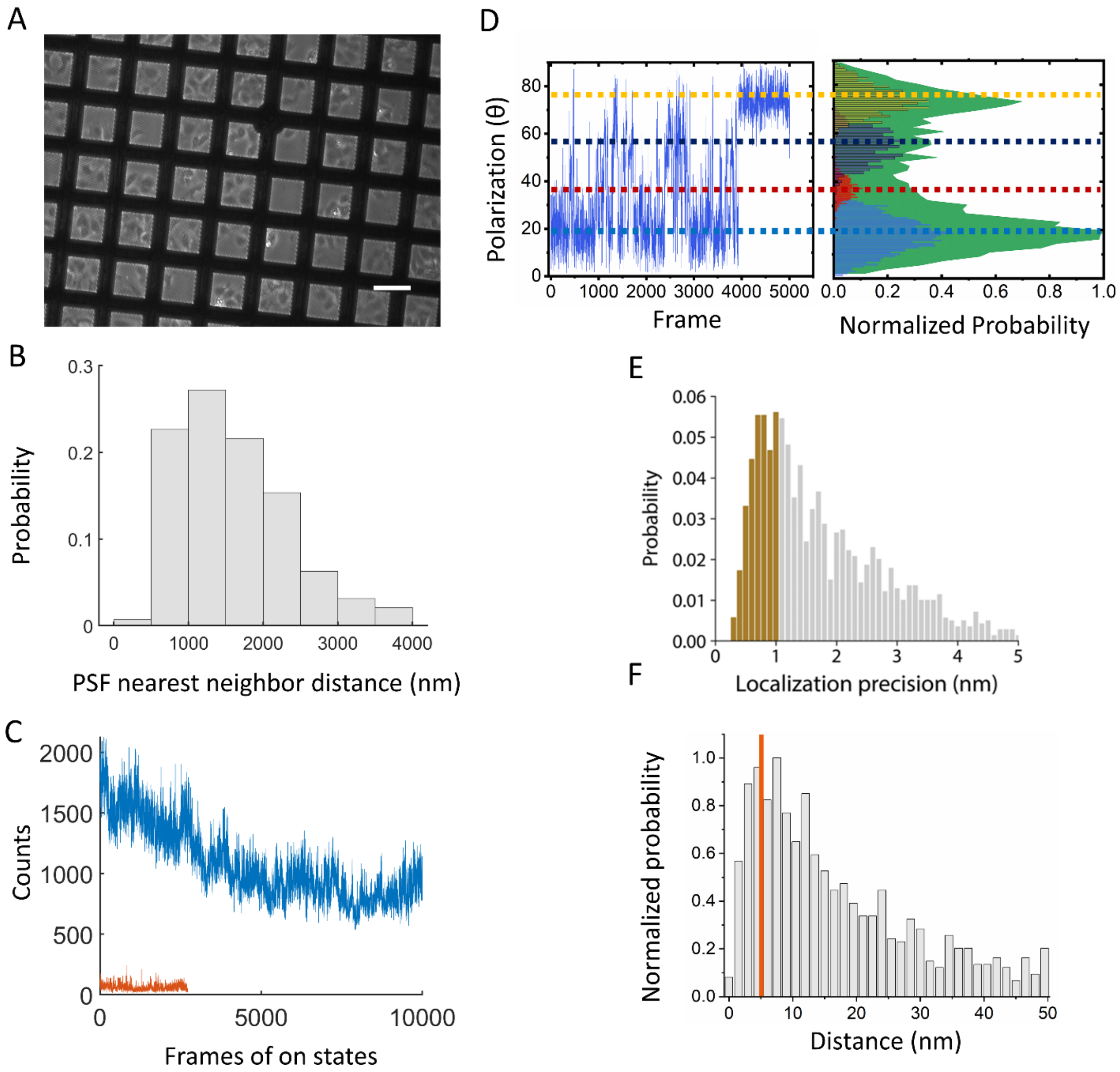

Fig. S5. Using polarization to identify individual fluorophores inside a PSF. (A) Brightfield image of the HL-1 cells grown onto UltrAuFoil QF R2/2 EM grids coated with Gelatine-Fibronactine. Scale bar: 100 µm. (B) Nearest neighbor distance between the PSFs in the image shown in Figure 4B in the main text. (C) The intensity time trace of a PSF with a large number of molecules (blue curve) compared to the signal of a PSF with a single molecule (orange curve). The signal was divided by the average signal of a single molecule to estimate the number of molecules qualitatively. (D) The polarization traces were extracted from each PSF as demonstrated recently (2) and explained in *Materials and Methods*. The polarization traces (blue color) were fit with an optimized version of the DISC algorithm (3), to obtain the number of polarization states per PSF. The figure shows exemplary traces with 4 different polarization states (yellow, dark blue, red, and light blue lines). The right panel shows a histogram of the polarization signal (green histogram). The segmented histograms based on the model fit (yellow, dark blue, red, and light blue) demonstrate proper fits of the polarization traces. (E) Localization precision of molecules in clusters with 2 proteins per PSF. (F) Distance histogram of molecules with localization precision below 3 nm.

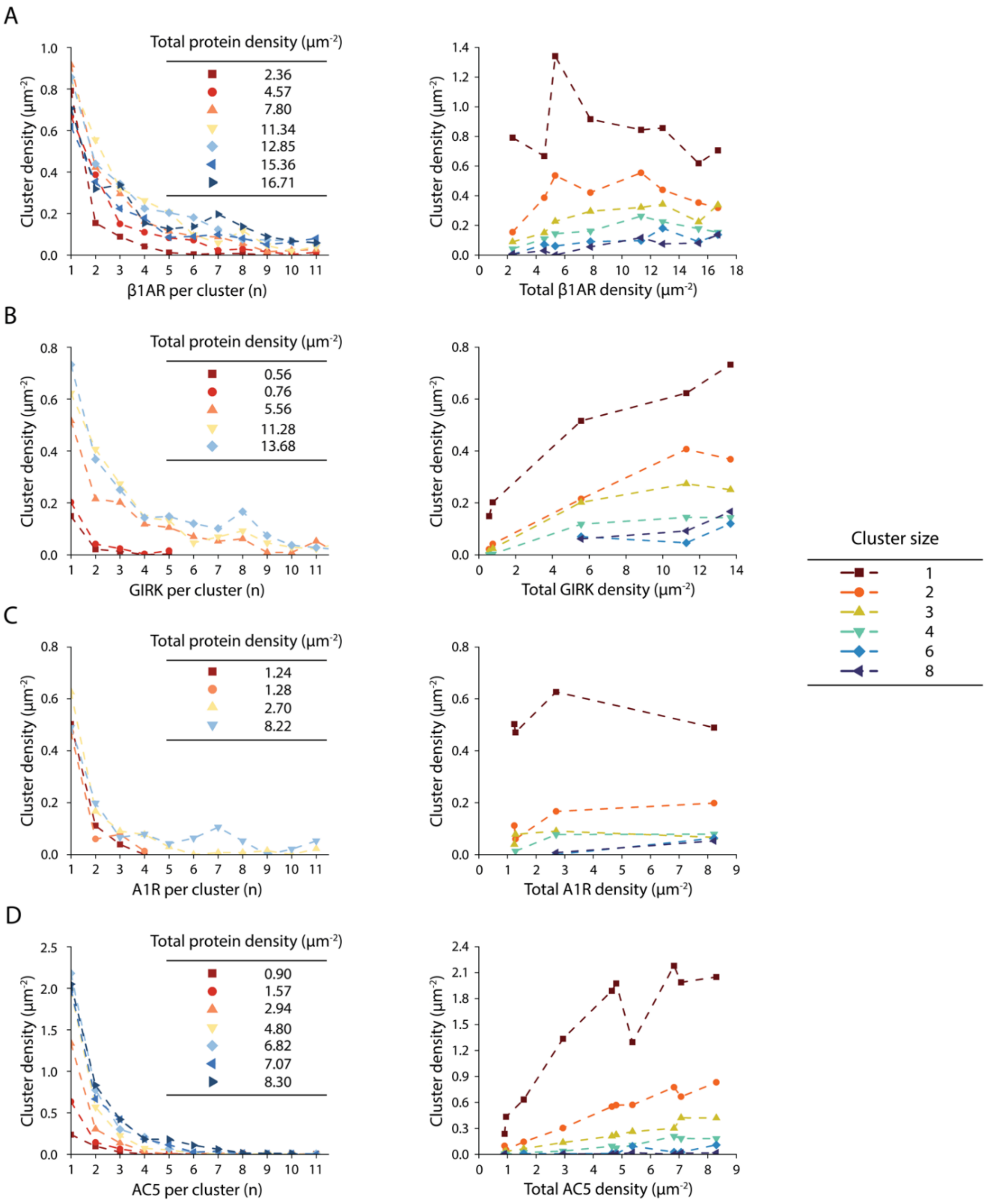

Fig. S6. Cluster size distributions of four membrane proteins expressed in CHO cells. (A) β1AR, (B) GIRK, (C) A1R, and (D) AC5 (adenylate cyclase 5) cluster size distributions (left) and cluster densities as a function of total protein density (right) in CHO cells heterologously expressing the proteins. Proteins are labeled with 18 nm gold particles as described in *Materials and Methods*. Non-specific gold particle labeling was estimated from CHO cells without heterologous expression and subtracted. Symbols show the data points. The dashed lines connect points. For β1AR, GIRK, and A1R the distributions appear to reach a critical distribution near the lowest total protein densities. But importantly, these distributions remain monotonically decreasing.

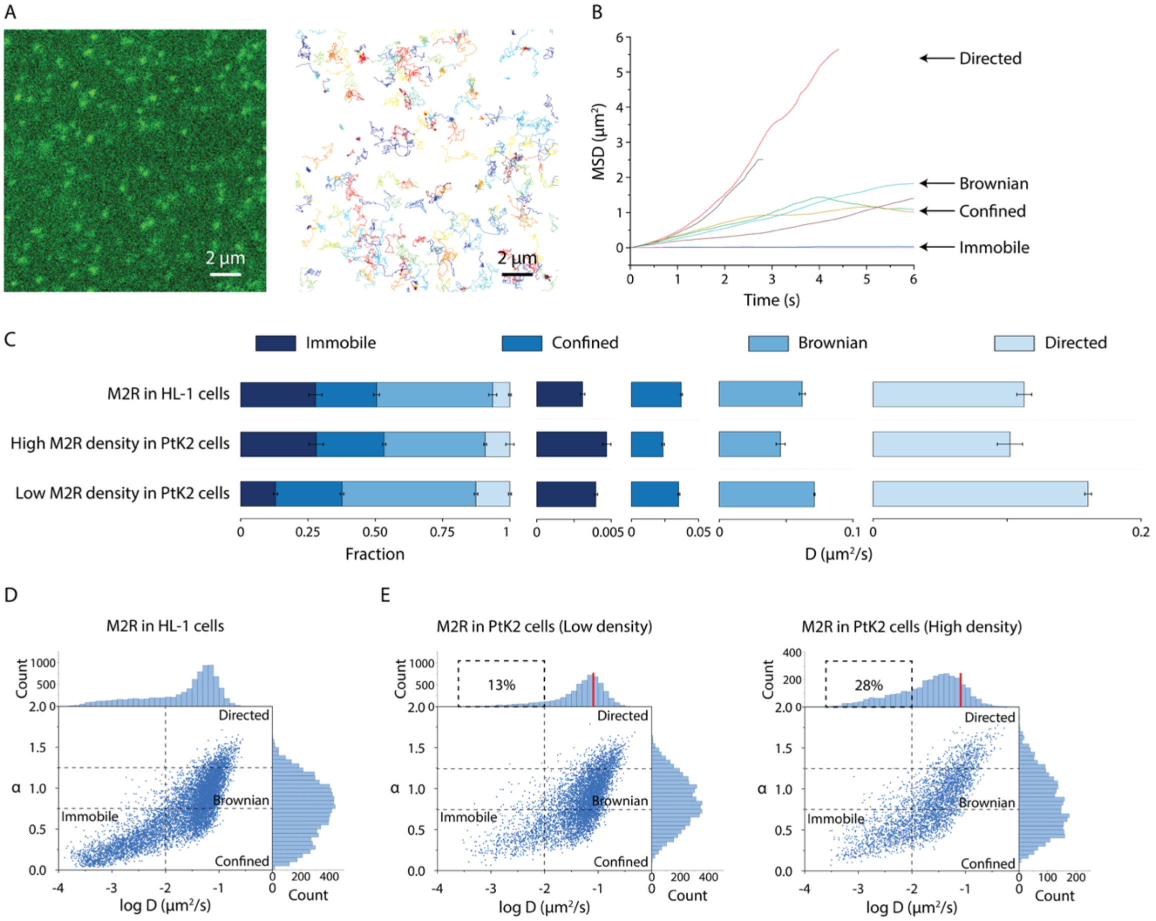

Fig. S7. Influence of expression level on diffusion of M2Rs in PtK2 cells. (A) A representative frame from a 70-second video recording of HA-M2R in PtK2 cells (left panel) and corresponding trajectories (right panel). HA-M2R was heterologously expressed and labeled with monoclonal anti-HA-Tag antibody conjugated to Alexa Fluor 488. Different colors indicate different trajectories. Scale bar: 2 µm. (B) The mean squared displacement (MSD) plot from representative M2R trajectories in PtK2 cells shows that M2Rs adopt different types of motion. (C) Fraction of M2R trajectories of different types of motion (right) and corresponding diffusion coefficients (right) under different conditions. Data expressed as means and standard errors from n trajectories. n = 6,652 for M2Rs in HL-1 cells, n = 6,868 for high M2R density in PtK2 cells, and n = 7,288 for low M2R density in PtK2 cells. (D) To compare the behavior of M2Rs in HL-1 and PtK2 cells we show a scatter plot and histograms of log D (diffusion coefficient, top) and α (anomalous diffusion exponent, right) for M2R trajectories in HL-1 cells. Dashed lines indicate cut-offs for classifying trajectories into four different types of motion. Trajectories with $D < 0.01$ µm^2/s were defined as immobile. Trajectories with $\alpha > 1.25$ were defined as directed. Trajectories with $0.75 \leq \alpha \leq 1.25$ were defined as Brownian. Trajectories with $\alpha < 0.75$ were defined as confined, as described previously (4). (E) M2R diffuses more slowly and has a higher probability of adopting immobile motion at higher density. Scatter plots and histograms of log D and α as in panel (D) for M2R trajectories in PtK2 cells. M2Rs are heterologously expressed to low (left) or high (right) densities. Cells with overlapping point spread functions in the first frame are defined as high-density cells, otherwise they are defined as low-density cells. For high-density cells, single particle detection and tracking are performed when most fluorophores are photobleached. Red lines in the top histograms indicate the peak position of log D in low-density cells. Dashed boxes indicate the fraction of immobile trajectories.

SI Materials and Methods

Antibodies

Antibodies used were anti-M2R (clone M2-2-B3, Millipore sigma, 1:200), GIRK4 (APC-027, alomone labs, 1:1500), β1AR (ab3442, abcam, 1:2000), AC 5/6 (PA5-75274, Invitrogen, 1:100), A1R (PA3-041, Invitrogen, 1:1000), HA-Tag antibody conjugated to Alexa Fluor® 488 (#28427, Cell Signaling Technology, 1:50), 18 nm Colloidal Gold AffiniPure™ Goat Anti-Rat IgG (H+L) (112-215-167, Jackson Immunoresearch, 1:20), 12 nm Colloidal Gold AffiniPure™ Goat Anti-Rat IgG (H+L) (112-205-167, Jackson Immunoresearch, 1:25), 18 nm Colloidal Gold AffiniPure™ Goat Anti-Rabbit IgG (H+L) (111-215-144, Jackson Immunoresearch, 1:20), 6 nm Colloidal Gold AffiniPure™ Goat Anti-Rabbit IgG (H+L) (111-195-144, Jackson Immunoresearch, 1:20), 40 nm Colloidal Gold AffiniPure™ Goat Anti-Rabbit IgG (H+L) (111-405-003, Jackson Immunoresearch, 1:20).

We chose to label GIRK4 because in cardiac cells, there are two subtypes: GIRK1 and GIRK4. GIRK4 can form both homomers and heteromers, whereas GIRK1 must form heteromers with GIRK4 to reach the cell membrane.

Molecular biology

Full-length human M2R (hM2R), hM2R with an N-terminal HA tag and GSSGGSSG linker (HA-M2R), mouse β1AR, mouse GIRK4, A1R and human AC5 were cloned into a pEG BacMam vector (5). Constructs were transformed into E. *coli* DH10Bac cells according to the manufacturer's instructions to generate bacmid containing hM2R or HA-M2R (Invitrogen). The bacmids were then transfected into Sf9 cells to produce baculoviruses with Cellfectin (Invitrogen). P3 viruses were obtained after two rounds of amplification.

Cell culture and protein expression

CHO cells (ATCC) were cultured at 37°C with 5% CO_2 in DMEM/F-12 medium supplemented with 10% fetal bovine serum. PtK2 cells (ATCC) were cultured at 37°C with 5% CO_2 in Eagle's Minimum Essential Medium supplemented with 10% fetal bovine serum. HEK cells (ATCC) were cultured at 37°C with 5% CO_2 in Dulbecco's Modified Eagle Medium supplemented with 10% fetal bovine serum and 2 mM L-glutamine. HL-1 cells (Sigma-Aldrich) were maintained according to the manufacturer's instructions. HL-1 cells were cultured at 37°C with 5% CO_2 in Claycomb medium supplemented with 10% HL-1 cell screened fetal bovine serum, 100 µg/ml penicillin/streptomycin, 0.1 mM norepinephrine and 2 mM L-glutamine. Cells were grown on T25 flasks coated with 0.005 mg/ml fibronectin and 0.02% gelatin solution. The supplemented medium was changed every day.

To seed cells for unroofing experiments, Quantifoil R1.2/21.2 300 mesh gold grids were glow discharged for 15 seconds. Grids were then washed three times with 70% EtOH, followed by four DPBS washes. To prepare grids for HL-1 cells, the grids were incubated with 0.005 mg/ml fibronectin and 0.02% gelatin solution at 37°C overnight. To prepare grids for CHO, PtK2, and HEK cells, grids were incubated in 0.1 mg/ml poly-D-lysine at room temperature for an hour, and then washed four times with DPBS. The grids were then incubated in 15 µg/ml laminin at 37°C for two hours, followed by four DPBS washes. Cells were then seeded onto the EM grids and grown to the desired confluency.

After cells reached 70% confluency, P3 viruses were added in a 1:10 (v:v) ratio and incubated at 37°C with 5% CO_2 for 16 hours to heterologously express proteins in CHO, PtK2, and HEK cells. The medium infected with virus was then replaced by fresh medium containing 10 mM sodium butyrate, and the temperature was lowered to 30°C. Experiments were conducted 24 hours after changing the temperature.

Detection of spontaneous calcium oscillations

Spontaneous calcium oscillations were detected according to the manufacturer's instructions (Sigma-Aldrich). HL-1 cells were seeded onto 35 mm glass bottom dishes (MatTeK) precoated with fibronectin/gelatin as described above and grown to confluency. Cells were rinsed with HEPES-buffered Tyrode's solution once and then incubated in Tyrode's solution with 5 µM Fluo-8 AM (AAT Bioquest) at 37°C for one hour. Cells were then washed twice with Tyrode's solution before being imaged at room temperature on an ECLIPSE Ti2 inverted microscope (Nikon) equipped with a ×40/0.65 objective lens and an Orca Fusion CMOS camera (Hamamatsu) using Nikon NIS-Elements AR5.4.1 software. Tyrode's solution with 10 µM isoproterenol or 10 µM carbachol was applied to cells to change the oscillation frequency.

Electrophysiology

HL-1 cells were split onto petri dishes four hours before the experiment. Currents were recorded in whole-cell mode using the pClamp software (Molecular Devices). Voltage was held at –60 mV and controlled with an Axopatch 200B amplifier (Molecular Devices). The current signal was low pass filtered at 1 kHz and digitized at 10 kHz with a Digidata 1440A digitizer (Molecular devices). For plotting purposes, data were reduced by a factor of 5. Pipettes were pulled to 3 – 5 MΩ resistance with a P-97 pipette puller (Sutter Instrument Company). A perfusion pencil was placed close to the recorded cell to locally change the extracellular solution. Before recording, cells were kept in a bath solution of 10 mM HEPES-KOH pH 7.4, 120 mM NaCl, 20 mM KCl, 2 mM $CaCl_2$ and 1 mM $MgCl_2$. During the recording, the extracellular solution was changed to either a high K^+ solution (10 mM HEPES-KOH pH 7.4, 80 mM NaCl, 60 mM KCl, 2 mM $CaCl_2$, 1 mM $MgCl_2$, 10 mM D-glucose) or a high K^+ solution supplemented with 10 µM carbachol. Pipettes were loaded with solution containing 10 mM HEPES-NaOH pH 7.4, 10 mM NaCl, 2 mM EGTA, 1 mM $MgCl_2$, 65 mM KF, 65 mM K-gluconate, 2 mM Mg-ATP, and 0.3 mM GTP.

Cell unroofing

The unroofing was performed as previously described with minor modifications (6). Grids seeded with cells were rinsed with DPBS (with calcium and magnesium) (Corning, 21-030-CV). Each grid was then picked up with a tweezer (Electron Microscopy Sciences) and dipped into a 2:1 ratio of pure water mixed with cytosol-like-buffer (CLB) containing 20 mM HEPES, 130 mM K gluconate, 5 mM NaCl, 2 mM $MgCl_2$ and pH 7.4, for 30 seconds. A 6 µl drop of undiluted CLB was added onto the cell side of the grid. The grid was immediately placed onto a filter paper, with the cell side facing the filter paper. The grid was removed from the filter paper once the buffer was entirely absorbed and the absorbent disk stopped growing. Grids were then rinsed three times with clean CLB. Whatman filter paper 2 (Sigma) was used for unroofing HL-1 and PtK2 cells, Whatman filter paper 40 (Sigma) was used for unroofing CHO cells, and Whatman filter paper 5 (Sigma) was used for unroofing HEK cells.

Immunolabeling and distribution analysis

After unroofing, grids were blocked with 3% goat serum diluted in DPBS with 1:100 protease inhibitor cocktail (Sigma, Catalog #: 539134) for 20 minutes. Primary antibody diluted in DPBS with 1% goat serum was then incubated with the grids for 1 hour or 1.5 hours (for AC) at room temperature. After three DPBS washes, the grids were then incubated with the secondary antibody conjugated to colloidal gold diluted in DPBS with 1% goat serum. After washing grids three times, they were then either stained with 1 % uranyl acetate or plunge frozen using a FEI Vitrobot Mark IV. The following parameters were used: 0 F, 4 second blotting time, and 0 second waiting time under 100% humidity and 22°C. A Teflon sheet was used on the side facing the membrane, and a standard blotting paper was used on the other side.
To quantify the protein distribution, EM micrographs were taken on Tecnai G2 Spirit BioTWIN Transmission Electron Microscope (ThermoFisher) with a pixel size of 1.898 nm or 200 keV Talos Arctica Transmission Electron Microscope (ThermoFisher) with a pixel size of 1.16 nm. Micrographs were stitched to form EM montages through SerialEM (7). Gold particle annotation and distribution analysis

were performed as previously described with minor modifications (8). Annotation of nanogold particles was carried out in Dragonfly (Dragonfly 2022.1 for Windows. Comet Technologies Canada Inc., Montreal, Canada; software available at https://dragonfly.comet.tech/) with a U-Net deep learning architecture, which was initially trained using manually annotated EM micrographs. The gold annotation outputs were then manually proofread to ensure accuracy. After annotation, the cluster size distribution and nearest neighbor distance analysis were conducted using the software Gold In-and-Out with a distance threshold of 28 pixels (8). Cluster centroids were calculated by averaging the x and y coordinates of all gold particles in each cluster.

Antibody screening

To identify the most effective antibody for each protein, six commercial antibodies were purchased for each target. Immunocytochemistry experiments were performed to determine which antibodies would bind specifically and efficiently to the target. In these experiments, CHO cells, with or without protein overexpression, were first fixed with 4% PFA for 10 minutes. Following fixation, cells were washed with DPBS and blocked with 3% goat serum and 0.1% Triton X-100 for 30 minutes. After additional washing with DPBS, primary antibodies at various concentrations, diluted in DPBS with 1% goat serum, were applied and incubated overnight at 4°C. The cells were then washed three times with DPBS and stained with fluorescent secondary antibodies before being imaged. Antibodies that produced strong fluorescence signals in the overexpression condition, and minimal signals in the negative controls, were selected for further testing. In the subsequent experiment, CHO cells with or without protein overexpression were grown on EM grids and unroofed as described above. The cells were stained with primary antibodies at different concentrations, followed by secondary antibodies conjugated to 18 nm gold particles. After washing, the samples were imaged under negative stain EM after uranyl acetate staining. The optimal antibody and concentration were determined based on high gold labeling in the overexpression samples and minimal gold labeling in the negative controls.

Note that the primary antibody labeling M2R is directed against a cytoplasmic-facing disordered loop approximately 170 amino acids in length, presumably enabling access to tightly packed M2R.

Tilt-series data collection and processing

The tilt series data collection and process were performed as previously described with minor modifications (9). The tilt series were collected on a 300 keV Titan Krios Transmission Electron Microscope (ThermoFisher) equipped with a Gatan Bioquantum energy filter (Gatan). Data were collected with a pixel size of 2.10 Å and a tilt range of −45° to 45° in 3° increments using SerialEM. The total dose was 126.5 $e^{-}/Å^{2}$ and the defocus was 2 µm. After full-frame alignment in MotionCor2, tilt series were coarsely aligned, refined with different alignment thicknesses, and reconstructed using Tomo3D SIRT in Appion-Protomo (10). Gold particles in tomograms were then manually annotated in 3DMOD.

Cryogenic light microscope imaging and analysis

For sample preparation, 1×10^{6} HL-1 cells were transferred to 3.5 cm µ-Dish (Ibidi) containing 8 UF EM grids with 2 nm carbon on top, facing upward. The cells adhered to the grids overnight. The following day, the cells were washed with Claycomb medium and then incubated with fresh medium containing 2 nM ATTO655-telenzepine fluorophore, kindly provided by Prof. Justin E. Molloy (The Francis Crick Institute), for 2-5 hours. Next, the cells were carefully washed using warm DPBS containing Ca^{2+} and Mg^{2+} and unroofed. After unroofing, the grids were then incubated in DPBS containing Ca^{2+}, Mg^{2+}, and 1:200 protease inhibitor cocktail and kept on a shaker for about 30 minutes to wash the cytosolic content. The cells were then washed three times with fresh CLB without protease inhibitor. Then, grids were plunge frozen using a Vitrobot with the parameters described above.

All imaging experiments were performed in a cryogenic microscope that is built around a Janis ST-500 flow cryostat and operates at liquid helium temperature (2). However, here we used an upgraded version of our cryogenic microscope which allows vitrified sample transfer in and out of the microscope at high-vacuum and liquid nitrogen temperature (11). The optical path is the same as in our previous work and can be found in cited papers (2, 12). Briefly, samples are loaded onto a cold finger and imaged by a 0.90NA microscope objective (MPLAN 100x, Mitutoyo), which is mounted in the cryostat vacuum chamber. The fluorescence signal is detected on two separate EMCCD cameras (Andor iXon) in a polarization-resolved configuration. For fast acquisition rates, the field of view was set to 211x313 pixels with a pixel size of 216 nm. The laser intensity used in all experiments was set to ~0.3 kW/cm^2, and images were recorded with 14-20 ms exposure times. For each field of view, we collected a total of 50,000-100,000 frames.

For image analysis, we analyzed raw image stacks from two polarization channels with custom-written MATLAB software as described in detail in two of our recent works (2, 12). The polarization time traces for each identified single point-spread function (PSF) were calculated using the intensity registered at each channel. These polarization time traces were analyzed in more detail by a routine that is based on the DISC algorithm (3) and a 2-dimensional Gaussian mixture model classification approach to find the number of polarization states per PSF. Since the dipole orientation polarization states of the fluorophores at 8 K are random but fixed, the number of identified polarization states within each PSF corresponds to the number of fluorophores per PSF/protein particle (2). This, in turn, allows us to annotate each fluorophore over time and localize it with high precision beyond the diffraction limit by clustering their coordinates accordingly. A 2-dimensional super-resolved image is then reconstructed by assigning a 2-dimensional Gaussian function to each localized polarization/fluorophore with a width given by the respective localization precision. These super-resolved images demonstrate different projections of the protein molecules within the sample. The 2-dimensional images are subjected to further analysis such as distance measurements. Given that the labeling efficiency is estimated to be 100% (13) (the dissociation constant of ATTO655-telenzepine is ~20 pM), the number of resolved polarization states reflects the number of M2R receptor proteins within a PSF. The calculated probability histogram of the number of M2R receptors per PSF is shown in Figure 4.

Single-particle tracking and colocalization time analysis

To track M2R in HL-1 cells with fluorescence, HL-1 cells were seeded onto 35 mm glass bottom dishes coated with fibronectin/gelatin and grown to the desired confluency. Cells were then incubated with 1 nM Cy3B-telenzepine, kindly provided by Prof. Justin E. Molloy (The Francis Crick Institute), at 37°C for 30 minutes. Cells were rinsed with DPBS three times before imaging. To track HA-M2R in PtK2 cells with fluorescence, PtK2 cells were seeded onto 35 mm glass bottom dishes coated with poly-D-lysine. After protein expression through virus infection, the medium was replaced by fresh medium containing 1:50 diluted anti-HA antibody conjugated to Alexa Fluor® 488 fluorescent dye (Cell Signaling Technology). After one hour of incubation, cells were then washed three times with medium before imaging. Cells were imaged at room temperature on an ECLIPSE Ti2 inverted microscope (Nikon) equipped with a ×100/1.45 oil objective lens and an Orca Fusion CMOS camera (Hamamatsu) using Nikon NIS-Elements AR5.4.1 software with a frame rate of 10 Hz.

Single particle detection and tracking were performed in ImageJ software with the TrackMate plugin (14, 15). Only PtK2 cells with overlapping point spread functions in the first frame were defined as high-density cells, all others were defined as low-density cells. For high-density cells, single particle detection and tracking were performed when most fluorophores were photobleached. In ImageJ, single particles were detected using the Laplacian of Gaussian (LoG) detector and tracked with the Linear Assignment Problem (LAP) tracker. After tracking, mean squared displacement analysis was conducted in MATLAB using msdanalyzer (16). Anomalous diffusion exponents and diffusion coefficients were calculated by fitting the Log-Log plot of the mean squared displacement curves. Fits with R^2 values lower than 0.5 were discarded.

The colocalization time analysis was performed as previously described with minor modifications (17). Videos for HL-1 cells were cropped and tracked using the u-track software in MATLAB. The outputs were then further analyzed using Polytracker.

To track HA-M2R labeled with 40 nm gold particles in PtK2 cells, PtK2 cells were seeded onto 35 mm glass bottom dishes coated with poly-D-lysine. After expressing HA-M2R in PtK2 cells, the medium was replaced by fresh medium containing 1:50 diluted anti-HA-Tag antibody conjugated to Alexa Fluor® 488 fluorescent dye (Cell Signaling Technology). After 1.5 hours in incubation, cells were washed with medium three times and incubated with 1:20 diluted 40 nm-gold-conjugated goat anti-rabbit secondary antibody (Jackson Immunoresearch) for 1 hour. Cells were then washed with medium followed by DPBS before imaging. Cells were imaged using DIC microscopy at room temperature on an ECLIPSE Ti2 inverted microscope (Nikon) equipped with a ×100/1.45 oil objective lens and an Orca Fusion CMOS camera (Hamamatsu) using Nikon NIS-Elements AR5.4.1 software with a frame rate of 33 Hz.

Expression and purification of GIRK and Kv2.1

Full-length mouse GIRK containing a C-terminal ALFA peptide and PPX-cleavable GFP was used as described in reference (18). Full-length human Kv2.1 with a C-terminal PreScission Protease (PPX)-cleavable GFP tag was used as described in reference (19). Both proteins were expressed in HEK293S GnTI$^-$ cells (ATCC) at a density of ~3×10^6 cells/mL infected with 12% (v/v) P3 baculovirus. Protein expression was induced by adding 10 μM sodium butyrate 8-10 hours after infection, and the incubation temperature was changed to 30°C for the duration of expression (48 hours). Cell pellets were harvested about 48 hours after induction and flash frozen in liquid nitrogen for later use.

Protein was purified as previously described (19). In brief, cells were lysed using a Dounce homogenizer and crude membranes were pelleted by centrifugation. Proteins were solubilized from membranes using 1.5% n-nodecyl-β-D-maltopyranoside (DDM) and purified using a GFP nanobody-coupled Sepharose resin (20). The GFP tag was cleaved using PPX and the protein was purified to homogeneity using size-exclusion chromatography with a Superose 6 Increase column (10/300GL), preequilibrated with SEC buffer (10 mM Tris pH 8.0, 100 mM KCl, 50 mM NaCl, 0.025% DDM and 5 mM DTT). Fractions containing protein were pooled and concentrated to ~1-2 mg/ml (A_{280}) for reconstitution.

Reconstitution of GIRK and Kv2.1 and grid preparation

POPC (PC 16:0 18:1, Avanti Polar Lipids) was dried to a thin film from chloroform using a gentle stream of Argon gas followed by a room temperature vacuum desiccator (19). The lipid film was resuspended at a concentration of 10 mg/mL by gentle vortexing in reconstitution buffer (10 mM Tris pH 8.0, 100 mM KCl, 50 mM NaCl) and small unilamellar vesicles were formed by bath sonication (Branson Ultrasonics M1800) until the solution was mostly transparent. DDM was added at a final concentration of 0.5% (5 mg/ml) to destabilize the vesicles. Purified protein was then mixed with the destabilized vesicles at protein-to-lipid ratios of ~1:20-1:30 (wt/wt) and the mixture was diluted using reconstitution buffer to a final lipid concentration of 3-5 mg/ml. Then, detergent was removed by dialyzing against 2 L of dialysis buffer (10 mM Tris pH 8.0, 100 mM KCl, 50 mM NaCl, 2 mM DTT and 1 mM EDTA) using a 50 kDa membrane (Spectra/Por 6 pre-wetted RC tubing). The dialysis buffer was changed twice each day until completion (4-5 days). The proteoliposomes were harvested and used immediately for grid preparation.

Negative-stain grids were prepared as follows. The proteoliposomes were diluted 1:10 in reconstitution buffer. EM grids (EMS CF400-CU: Carbon Film, 400 Mesh, Copper) were glow-discharged for 15 seconds. 5 μL of diluted sample was applied to the grid and incubated for 1 minute. After blotting the sample away, the grid was washed with distilled water and then the sample was stained by applying a saturated solution of uranyl acetate three times. Excess solution was blotted away after each application.

Cryo-EM grids were prepared as follows. 3.5 μL of the proteoliposome suspension was applied onto a glow-discharged Quantifoil R1.2/1.3 400 mesh Au grid and incubated for 3 minutes at 20°C under 100%

humidity. Excess solution was blotted away manually with filter paper, another 3.5 µL of sample was applied for 20 seconds, and then the grid was blotted for 3 seconds with a blotting force of 0 and flash frozen in liquid ethane using a FEI Vitrobot Mark IV (FEI).

Electron microscopy of GIRK and Kv2.1 and data analysis

Negative-stain transmission electron microscopy (EM) images to quantify protein distribution were acquired on a Tecnai G2 Spirit BioTWIN operating at 120 kV using a magnification of 30,000x (0.304 nm/px). TIF files were exported, and particles were manually picked using the software Fiji (21). Fiji was also used to generate masks to define the areas of the membrane sheets. The software Gold In-and-Out (8) was used to obtain cluster size distributions for each micrograph using the particle locations. The cluster size data from 5-10 images (including 10-30 membrane sheets in total for each protein) were combined and plotted for each protein as a distribution using OriginPro.

Cryogenic electron microscopy (cryo-EM) data were collected on a 300-keV FEI Titan Krios1 microscope equipped with a Gatan K3 camera. Approximately 2500 micrographs were collected using a pixel size of 0.1376 nm/px, target defocus range of −1.0 to −2.5 µm, and a total dose of ~60 $e^{-}/Å^{2}$. Data processing was carried out using cryoSPARC v4.1.2 as previously described (19). Particles were picked using a TOPAZ picking model on motion- and CTF-corrected micrographs.

2-dimensional classification of top and bottom views were used to separate particles on the top membrane and on the bottom membrane by using the handedness of the class averages. The x,y coordinates of particles (on one membrane) were exported, and a Python script (22) was written to analyze the cluster size distribution for selected micrographs that had membrane spanning the whole image. To define the size of the clusters, we used the Agglomerative Clustering algorithm from Scikit-Learn (http://jmlr.org/papers/v12/pedregosa11a.html), which uses a set distance cutoff (for GIRK, 15 nm was used) between particles to cluster them. The cluster size information was pooled from the 20 micrographs selected for analysis and plotted in OriginPro.

Expression, purification, and reconstitution of M2R-ALFA for freestanding bilayer imaging

The human muscarinic 2 receptor (hM2R) modified to include a C-terminus ALFA-tag, a 3C PreScission Protease recognition site, enhanced yellow fluorescent protein (eYFP), and a polyhistidine tag (His10-tag) was expressed and purified as previously described (23). This construct was cloned into a pFastBac vector for expression using the Bac-to-Bac baculovirus system in Sf9 insect cells. The virus was amplified and used for large-scale expression. Cells were cultured, infected, and harvested, then resuspended in phosphate-buffered saline, frozen, and stored. Cells were lysed by osmotic shock, and membrane proteins were extracted using 1% DDM:0.01% CHS. The supernatant was bound to TALON resin, washed with buffers of increasing NaCl concentration, and eluted with imidazole. Eluted proteins were diluted and re-bound to GFP nanobody-coupled Sepharose resin for buffer exchange. The protein underwent buffer exchange by washing the resin on a gravity column with 20 column volumes (CVs) of buffer. This buffer contained the same components as the dilution buffer but had an increased iperoxo concentration of 50 µM. 3C PreScission Protease was added to the resin, and the protein was incubated overnight to ensure complete digestion, and to replace the ligand bound to M2R from the antagonist atropine to iperoxo. The cleaved M2R was eluted by gravity flow-through collection of the GFPNb resin. M2R constructs were dephosphorylated and concentrated using 30-kDa molecular weight cutoff (MWCO) concentrators before undergoing purification by size exclusion chromatography on a Superdex 200 Increase 10/300 GL column with a running buffer containing 20 mM Hepes (pH 7.4), 100 mM KCl, 50 mM NaCl, 10 µM iperoxo, 100 µM TCEP, and 0.1%:0.01% DDM:CHS. M2R reconstitution in lipid vesicles was performed immediately after gel filtration. Non-reconstituted purified M2R was flash-frozen with 10% glycerol and stored at −80°C.

M2R-ALFA was incorporated into lipids at a 3:1 weight ratio of POPE to POPG. After drying the lipids with liquid nitrogen and vacuum, the lipid film was rehydrated in a buffer consisting of 20 mM Hepes (pH 7.4),

100 mM KCl, 50 mM NaCl, 10 µM iperoxo, and 100 µM TCEP to obtain a stock concentration of 10 mg/ml. The POPE:POPG (3:1) lipids were sonicated until clear in a water bath sonicator. To promote protein insertion into vesicles, 1% n-decyl-β-maltoside (DM) was added to the lipids. The lipids were gently nutated for 30 minutes at room temperature and then sonicated again. To achieve a protein-to-lipid ratio (PLR) of 1:20, M2R-ALFA and POPE:POPG lipids were combined to final concentrations of 0.25 mg/ml and 5 mg/ml, respectively. The mixture was then nutated for 1 hour at room temperature. To remove the detergent, biobeads (Bio-Beads SM-2 Adsorbent, Bio-Rad) equilibrated in 20 mM Hepes (pH 7.4), 100 mM KCl, 50 mM NaCl, 10 µM iperoxo, and 100 µM TCEP were added to the protein-lipid mixture so that the dry volume of beads was approximately one-third the volume of the vesicles. The mixture was then nutated at 4°C, with biobeads being replaced every 8 to 12 hours for a total of 3 to 4 changes. After detergent removal, the proteoliposomes (PLs) were collected, flash-frozen, and stored at −80°C until use.

Freestanding bilayer microscope (FBM) experiments and analysis

Detailed protocols on how to use and build the FBM and perform experiments have been previously published (23). After M2R proteoliposome fusion on a freestanding lipid bilayer, illumination was followed by the addition of 1 nM labeled ALFA nanobody (NbALFA) for single molecule labeling, and an additional 5 ml of perfusion to wash out the unbound NbALFA. The illuminated area for each bilayer was adjusted by changing the input voltage amplitude on a galvo scanner. Videos were then recorded at a frame rate of 50 Hz. Single-particle tracking (SPT) was performed using u-track software. Interactions between M2Rs were analyzed as described before for cell-based imaging. Further data analysis was conducted using custom-written software in MATLAB (MathWorks).

Fitting analytical expressions to cluster size distributions

When fitting single data sets (Figure 9), Eq. 5 was fit to cluster size distributions under the constraint in Eq. 8 set by the measured total concentration of labels. The measured monomer concentration c_{mon} and total concentration c_{tot} were entered, $\Delta G^0_{\substack{mon\ to \\ bulk}}$ fit, and A_0 calculated. When globally fitting multiple data sets, the fitting procedure was carried out on simulated (Figure 7) or experimental (Figure 8) monomer-normalized distributions with Eqs. 5 and 8 normalized by c_{mon}. A single value of $\Delta G^0_{\substack{mon\ to \\ bulk}}$ was fit for all curves. Then A_0 was calculated for individual data sets using the monomer-normalized constraint equation and c_{tot} normalized by c_{mon} for each data set. For the global fitting, monomer normalization ensured that each curve provided a similar weight to the determination of $\Delta G^0_{\substack{mon\ to \\ bulk}}$ across data sets. All calculations and fits were carried out in Mathematica (Wolfram Research, Inc., Mathematica, Version 14.1, Champaign, IL (2024)).

Simulations

Simulations of diffusing particles interacting on a grid (Figure 7 and SI Appendix, Movie S5) were written in Mathematica (Wolfram Research, Inc., Mathematica, Version 14.1, Champaign, IL (2024)). The code is available on the MacKinnon Lab website.

Graphs and figures were produced using Origin(Pro), Version 2023 (OriginLab Corporation, Northampton, MA, USA.) and Adobe Illustrator (Adobe Inc. 2021).

Movies S1-S4 (separate files). Representative movies of spontaneous calcium oscillations in confluent HL-1 cells. Confluent HL-1 cells are loaded with Fluo-8 AM and imaged. Spontaneous calcium oscillations were observed at basal level (Movie S1), accelerated by isoproterenol (Movie S2) and slowed by carbachol (Movie S3) and adenosine (Movie S4). 10 μM isoproterenol, 10 μM carbachol, and 10 μM adenosine were used.

Movie S5 (separate file). Diffusion of interacting particles on a grid. 200 particles undergo a random walk on a 100 x 100 (10,000 site) 2-dimensional grid such that two particles cannot occupy the same site. As the slide bar is moved, if a particle has at least one neighbor, the probability it will not step to a neighboring site (if unoccupied by a particle) is increased by a constant weight. The dimensional edge length is assigned through real distance per integer unit of grid length (rpg) and time per frame by $\frac{(rpg)^2}{4D}$, where D is the diffusion coefficient. Code is available at (MacKinnon Lab website). We emphasize that the simulations considered here are not based on an *nmer* energy function (see Appendix 3), and are not set up to examine the equilibrium (Boltzmann) HOTS distribution (24). The toy model serves to illustrate, in very simple terms, how particles can self-assemble into clusters through self-recognition.

Movie S6 (separate file). Representative movie of 40 nm gold-labeled HA-M2R in PtK2 cells. HA-M2Rs were labeled with a fluorescent antibody, then sparsely labeled with a secondary antibody containing a single 40 nm gold particle and imaged under differential interference contrast microscopy. The video was acquired at 6.6 frames/s and lasted 10 min. Fluorescent images were taken before and after the video was recorded to ensure that large puncta remained relatively stationary. The gold movie was superimposed on a fluorescent image of the same region of the membrane. The movie is accelerated four times.

SI References

1. H. A. Bent, *The Second Law: An Introduction to Classical and Statistical Thermodynamics* (Oxford University Press, 1965).
2. H. Mazal, F. F. Wieser, V. Sandoghdar, Deciphering a hexameric protein complex with Angstrom optical resolution. *eLife* **11** (2022).
3. D. S. White, M. P. Goldschen-Ohm, R. H. Goldsmith, B. Chanda, Top-down machine learning approach for high-throughput single-molecule analysis. *eLife* **9**, e53357 (2020).
4. T. Sungkaworn *et al.*, Single-molecule imaging reveals receptor–G protein interactions at cell surface hot spots. *Nature* **550**, 543-547 (2017).
5. A. Goehring *et al.*, Screening and large-scale expression of membrane proteins in mammalian cells for structural studies. *Nature Protocols* **9**, 2574-2585 (2014).
6. C. F. Peitsch, S. Beckmann, B. Zuber, iMEM: Isolation of Plasma Membrane for Cryoelectron Microscopy. *Structure* **24**, 2198-2206 (2016).
7. D. N. Mastronarde, Automated electron microscope tomography using robust prediction of specimen movements. *Journal of Structural Biology* **152**, 36-51 (2005).
8. D. Guerrero-Given *et al.*, Gold In-and-Out: A Toolkit for Analyzing Subcellular Distribution of Immunogold-Labeled Membrane Proteins in Freeze-Fracture Replica Images. *Front Neuroanat* **16**, 855218 (2022).
9. C. A. Haselwandter, Y. R. Guo, Z. Fu, R. MacKinnon, Quantitative prediction and measurement of Piezo's membrane footprint. *Proc Natl Acad Sci U S A* **119**, e2208027119 (2022).
10. A. J. Noble, S. M. Stagg, Automated batch fiducial-less tilt-series alignment in Appion using Protomo. *J Struct Biol* **192**, 270-278 (2015).
11. H. Mazal, F.-F. Wieser, D. Bollschweiler, A. Schambony, V. Sandoghdar, Cryogenic light microscopy with sub-nanometer resolution deciphers the structural conformations of PIEZO1. *In preparation* (2024).
12. D. Böning, F.-F. Wieser, V. Sandoghdar, Polarization-Encoded Colocalization Microscopy at Cryogenic Temperatures. *ACS Photonics* **8**, 194-201 (2021).
13. T. A. Nenasheva *et al.*, Abundance, distribution, mobility and oligomeric state of M_2 muscarinic acetylcholine receptors in live cardiac muscle. *J Mol Cell Cardiol* **57**, 129-136 (2013).
14. J.-Y. Tinevez *et al.*, TrackMate: An open and extensible platform for single-particle tracking. *Methods* **115**, 80-90 (2017).
15. D. Ershov *et al.*, Bringing TrackMate into the era of machine-learning and deep-learning. *bioRxiv* 10.1101/2021.09.03.458852, 2021.2009.2003.458852 (2021).
16. N. Tarantino *et al.*, TNF and IL-1 exhibit distinct ubiquitin requirements for inducing NEMO–IKK supramolecular structures. *Journal of Cell Biology* **204**, 231-245 (2014).
17. J. Moller *et al.*, Single-molecule analysis reveals agonist-specific dimer formation of micro-opioid receptors. *Nat Chem Biol* **16**, 946-954 (2020).
18. M. E. Falzone, R. MacKinnon, Gβγ activates PIP2 hydrolysis by recruiting and orienting PLCβ on the membrane surface. *Proc Natl Acad Sci U S A* **120**, e2301121120 (2023).
19. V. S. Mandala, R. MacKinnon, The membrane electric field regulates the PIP(2)-binding site to gate the KCNQ1 channel. *Proc Natl Acad Sci U S A* **120**, e2301985120 (2023).
20. A. Kirchhofer *et al.*, Modulation of protein properties in living cells using nanobodies. *Nature Structural & Molecular Biology* **17**, 133-138 (2010).
21. J. Schindelin *et al.*, Fiji: an open-source platform for biological-image analysis. *Nature Methods* **9**, 676-682 (2012).
22. C. R. Harris *et al.*, Array programming with NumPy. *Nature* **585**, 357-362 (2020).
23. G. Perez-Mitta, Y. Sezgin, W. Wang, R. MacKinnon, Freestanding bilayer microscope for single-molecule imaging of membrane proteins. *Sci Adv* **10**, eado4722 (2024).
24. D. Frenkel, B. Smit, *Understanding Molecular Simulation: From Algorithms to Applications* (Academic Press, 2023).
